# The transcriptome and miRNome profiling of glioblastoma tissues and peritumoral regions highlights molecular pathways shared by tumors and surrounding areas and reveals differences between short-term and long-term survivors

**DOI:** 10.18632/oncotarget.4151

**Published:** 2015-06-01

**Authors:** Barbara Fazi, Armando Felsani, Luigi Grassi, Anna Moles, Daniel D'Andrea, Nicola Toschi, Daria Sicari, Pasquale De Bonis, Carmelo Anile, Maria Giovanna Guerrisi, Emilia Luca, Maria Giulia Farace, Giulio Maira, Silvia Anna Ciafré, Annunziato Mangiola

**Affiliations:** ^1^ Department of Biomedicine and Prevention, University of Rome “Tor Vergata”, Rome, Italy; ^2^ CNR, Institute of Cell Biology and Neurobiology, Rome, Italy; ^3^ Genomnia srl, Lainate, Milan, Italy; ^4^ Department of Physics, University of Rome “La Sapienza”, Rome, Italy; ^5^ Department of Radiology, Athinoula A. Martinos Center for Biomedical Imaging, Boston, MA, USA; ^6^ Harvard Medical School, Boston, MA, USA; ^7^ Department Head and Neck, Institute of Neurosurgery, Catholic University of Sacred Heart, Rome, Italy; ^8^ Neurosurgery, Ferrara University Hospital S. Anna, Cona di Ferrara, Ferrara, Italy; ^9^ Institute of Anatomic Pathology, University Hospital “A. Gemelli”, Catholic University of Sacred Heart, Rome, Italy

**Keywords:** glioblastoma, microRNA, TGFβ, peritumoral area, editing

## Abstract

Glioblastoma multiforme (GBM) is the most common and deadliest primary brain tumor, driving patients to death within 15 months after diagnosis (short term survivors, ST), with the exception of a small fraction of patients (long term survivors, LT) surviving longer than 36 months. Here we present deep sequencing data showing that peritumoral (P) areas differ from healthy white matter, but share with their respective frankly tumoral (C) samples, a number of mRNAs and microRNAs representative of extracellular matrix remodeling, TGFβ and signaling, of the involvement of cell types different from tumor cells but contributing to tumor growth, such as microglia or reactive astrocytes. Moreover, we provide evidence about RNAs differentially expressed in ST vs LT samples, suggesting the contribution of TGF-β signaling in this distinction too. We also show that the edited form of miR-376c-3p is reduced in C *vs* P samples and in ST tumors compared to LT ones. As a whole, our study provides new insights into the still puzzling distinction between ST and LT tumors, and sheds new light onto that “grey” zone represented by the area surrounding the tumor, which we show to be characterized by the expression of several molecules shared with the proper tumor mass.

## INTRODUCTION

Glioblastoma multiforme (GBM; World Health Organization [WHO] grade IV) is the most common and most malignant primary tumor of the brain. Excessive proliferation, diffuse infiltration into surrounding brain tissue and suppression of antitumor immune surveillance contribute to the malignant phenotype of glioblastomas. Despite multimodal aggressive treatment, the prognosis of GBM patients is poor and the median survival time after diagnosis is still in the range of just 12 months [1], or even shorter [2]. An interesting exception to this rule is a small fraction (less than 10%) of GBM patients, referred to as long-term survivors (LTS), who survive longer than 36 months. Several attempts have been made to unravel the molecular differences at the basis of such a clinically relevant issue, addressing either the genomic aspects [3] or the mutation or methylation state of specific genes [4, 5]. Some studies also performed genome-wide gene expression analyses by using the microarray technology, resulting in the discovery of differentially expressed genes [6]. Nonetheless, we are still far from the comprehension of the reasons leading a glioblastoma patient to survive significantly longer than another one.

Targeted therapies have been introduced for GBM, based on information obtained from molecular studies of the tumor tissue [7]. Nevertheless, no clear survival benefit has been demonstrated, probably because tumor tissue represents the last step of tumorigenesis involving a number of alterations allowing tumor cells to survive. Since recurrence occurs in peritumoral tissue in about 95% of patients [8], getting a deeper insight into the biology of the peripheral areas immediately surrounding tumors is of great interest, as it may unveil molecular alterations representing the first signs of the future malignant evolution to glioblastoma. In this regard, only a few works have addressed the question of which molecular alterations affect the brain area surrounding the tumor [9, 10]. These works have indicated that peritumoral areas are diverse and composed of several different cellular components, such as microglia, reactive astrocytes, T lymphocytes, and others [11]; at the same time, they have started showing that the peritumoral areas are deeply interested by molecular and metabolic changes rendering them very different from normal brain and much closer to transformed cells, or at least necessary to sustain the growth of the frankly tumor cells.

In this study, we report a broad analysis of central tumor samples, from both long term survivors (LT) and short term ones (ST), integrated by the same analysis performed on peritumoral areas from the same patients. We provide data from gene expression (SAGE) analysis and from microRNA deep sequencing that, as a whole, allow not only to draw a novel comprehensive picture of LT vs ST tumors, but also to shed light into the critical, tumor-supporting peritumor areas.

## RESULTS

### Gene expression (SAGE) analysis of GBM centers and peritumor areas reveals RNA molecules differentially expressed in all tumor centers vs their own peritumorareas

We collected frankly tumoral areas (C) as well as peritumoral areas from 4 long term (LT: survival longer than 36 months) and 9 short term (ST: survival shorter than 36 months) patients diagnosed with primary glioblastoma (Table 1a). The peritumoral samples (P) were collected at an average distance of 2 cm from the border of the enhanced tumor, and did not show any evidence of tumor presence at macroscopic evaluation performed by the surgeon. We submitted total RNAs extracted from these samples to SAGE profiling, and, for each C and P sample in our SAGE dataset, we determined the GBM subtype by calculating the single sample Gene Set Enrichment Analysis [12] for each SAGE profile relative to the classified gene lists from Verhaak et al. [13]. Single-sample GSEA (ssGSEA), an extension of Gene Set Enrichment Analysis (GSEA), calculates separate enrichment scores for each pairing of a sample and gene set. Each ssGSEA enrichment score represents the degree to which the genes in a particular gene set are coordinately up- or down-regulated within a sample. C samples were largely heterogeneous, showing correlation with neural (5/13 samples), mesenchymal (4/13 samples), classical (2/13 samples), or proneural (2/13 samples) subtypes. In contrast, the majority (8/13) of samples from P regions showed the highest correlation with the neural subtype. No specific enrichment in any subtype was observed when comparing the ST with the LT samples (Table 1b).

**Table 1a:** Characteristics of patients analyzed in this study

Patient	Sex	Age	Survival (months)	Peritumoral infiltration	SAGE	miRNome
**Long term**						
**LT1**	M	34	60	Yes 10%	X	X
**LT2**	F	29	54	no	X	X
**LT3**	F	71	36	no	X	X
**LT4**	F	46	53	Yes 30%	X	
**Short term**						
**ST2**	F	62	15	Yes 20%	X	X
**ST4**	M	73	6	no	X	X
**ST5**	F	70	13	no	X	
**ST7**	M	54	15	Yes 30%	X	X
**ST8**	M	51	30	Yes 30%	X	X
**ST9**	M	64	17	no	X	
**ST10**	F	66	16	Yes 5%	X	X
**ST11**	M	67	13	no	X	X
**ST12**	M	70	19	Yes 30%	X	X

All patients were diagnosed of primary glioblastoma. The degree of infiltration in the peritumor areas was calculated as in ref. 70. The last two columns indicate which samples underwent SAGE and miRNome analysis, respectively.

**Table 1b:** Classification of C and P samples according to Verhaak et al. [13] from single sample gene set enrichment analysis of SAGE datasets

	C	P
**Long term**		
**LT1**	PN	NL
**LT2**	NL	MES
**LT3**	NL	CL
**LT4**	CL	NL
**Short term**		
**ST2**	NL	NL
**ST4**	PN	NL
**ST5**	NL	CL
**ST7**	MES	MES
**ST8**	MES	NL
**ST9**	NL	NL
**ST10**	MES	NL
**ST11**	MES	NL
**ST12**	CL	CL

In order to investigate which RNA molecules can distinguish frank tumor areas (Cs) from the corresponding P regions, we analyzed our SAGE data for differentially expressed molecules. Filtering these results for those RNAs whose differential expression was characterized by a FDR < 0.05, yielded the transcripts listed in Suppl. Tables 1a and 1b. Among RNAs overexpressed in tumor centers *vs* peritumoral areas, only four were shared by STCs and LTCs (compare Suppl. Table 1a and 1b), while other molecules were specific of either STCs or LTCs. For example, STCs exhibited, compared to STPs, the overexpression of several markers of the mesenchymal subtype of glioblastoma [13], such as COL1A1, COL1A2, COL5A1, IGFBP6, DCBLD2, many of which are also typically expressed by microglia or reactive astrocytes in glioblastoma microenvironment [10]. Conversely, RNAs overexpressed in LTCs vs LTPs showed an enrichment in markers of the neural subtype of glioblastoma, such as BASP1, CDC42, and SH3GL2 [13].

**Figure 1:**
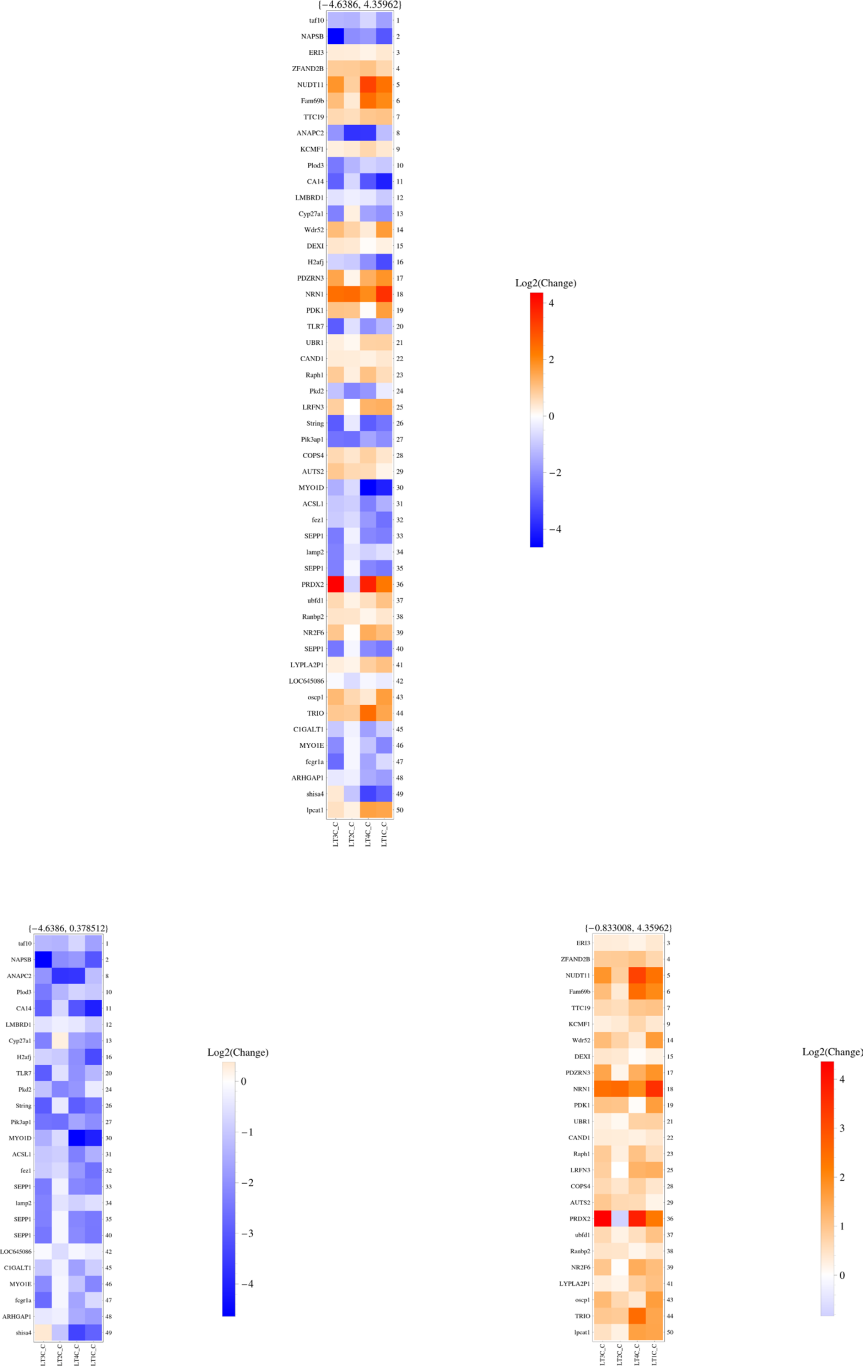
SAGE-based analysis of transcripts differentially represented in each pair of LTC vs LTP samples The heatmap shows the top 50 highest ranking RNA molecules, based on their “average merit”. The middle panel shows all 50 top RNA molecules, based on their average merit; in the left panel, only RNAs underexpressed in each LTC vs its own LTP are shown; in the right panel, only RNAs overexpressed in each LTC vs its own LTP are shown. The range of Log2 change is depicted in the vertical color bar on the right of each panel, and the corresponding numbers are written on top of each panel. The right side of each panel reports the ranking position of each RNA, whose name is illustrated on the left side of the panel.

**Figure 2:**
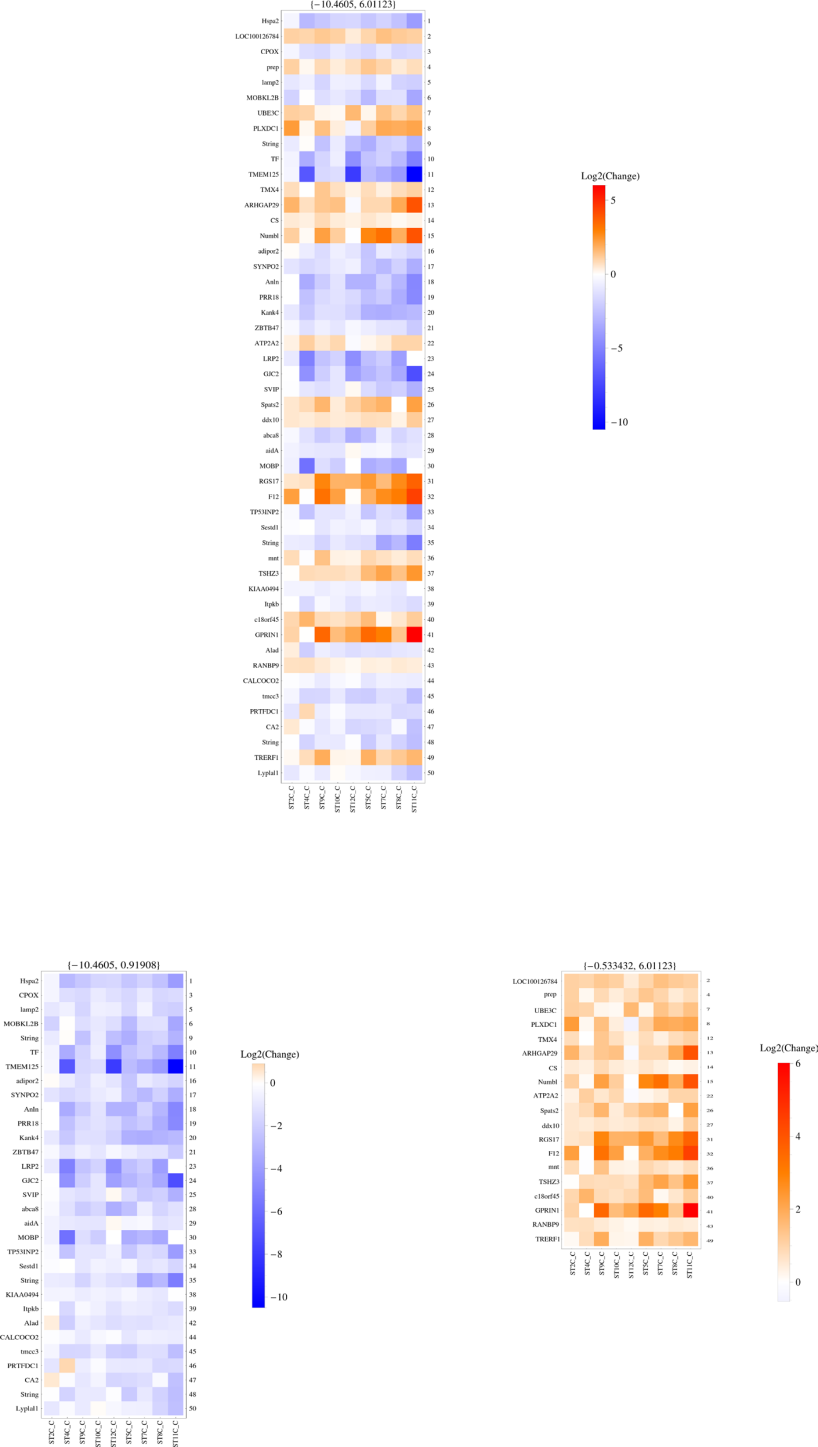
SAGE-based analysis of transcripts differentially represented in each pair of STC vs STP samples The heatmap shows the top 50 highest ranking RNA molecules, based on their “average merit”. The middle panel shows all 50 top RNA molecules, based on their average merit; in the left panel, only RNAs underexpressed in each STC vs its own STP are shown; in the right panel, only RNAs overexpressed in each STC vs its own STP are shown. The range of Log2 change is depicted in the vertical color bar on the right of each panel, and the corresponding numbers are written on top of each panel. The right side of each panel reports the ranking position of each RNA, whose name is illustrated on the left side of the panel.

We then asked a slightly different question, that is which RNAs are differentially expressed in each C *vs* P pair, so that their expression can distinguish each C sample when compared to its own P area. To this aim, we employed a ReliefF algorithm [14] in conjunction with leave-one-out cross validation to compute the average (over all validation folds) merit of every RNA molecule for the binary classification problem. Figures 1 and 2 show the heatmaps drawn based on average merit ranking for LT and ST comparisons, respectively. Among the 50 top ranking RNAs, both over-and under-expressed molecules (C vs P) were found in almost equal proportions. Interestingly, almost no RNAs were shared by LT C/P and ST C/P comparisons, with the exception of one noncoding isoform of ELOVL fatty acid elongase 1, which was more expressed in both STPs and LTPs when compared to their own tumor centers; however, amongst the mRNAs found to be overexpressed in both LTCs and STCs with respect to their P regions, several RNAs were seen to be involved in the same processes, such as those encoding for two nuclear pore complex proteins, RANBP2 and RANBP9 (overexpressed in LTCs and STCs) respectively, or UBE3C and UBR1, both members of the ubiquitin protein ligase E3 complex (overexpressed in STCs and LTCs) or TMX4 and PRDX2, involved in cell redox homeostasis (overexpressed in the majority of STCs and LTCs). Among RNAs differentially expressed in the ST group, but not in the LT one, we found the prolylendopeptidase (PREP), highly expressed in each STC compared to its own STP. The enzyme encoded by this gene is a serine protease working at the digestion of ECM by fibroblasts upon fibroblast activation, in all those processes mediated by cancer associated fibroblasts and supporting tumor development [15], such as tissue remodeling, angiogenesis [16], and immune tolerance [17]. Another interesting example is ARHGAP29, encoding for Rho-GTPAse Activating Protein 29 (overexpressed in STCs *vs* STPs but not in LTCs *vs* LTPs): this mRNA is part of a previously described “mesenchymal” signature of glioblastoma [13], also found to be expressed by reactive astrocytes and microglia [10]. On the LT side, we found a consistent overexpression of neuritin 1 (NRN1), a neurotrophin promoting neuronal migration [18] and induced by hypoxia [19] (in all LTCs vs their respective LTPs). Two mRNAs whose expression characterizes LTCs vs LTPs, LRFN3 (encoding for Leucine Rich repeat and Fibronectin type III domain containing 3) and NR2F6 (Nuclear Receptor subfamily 2 group F member 6), were previously included in the “classical” signature of glioblastoma [13].

Taken together, these findings indicate that GBM centers show a different expression profile compared to peritumor areas, and that the mRNAs whose expression is relevant to distinguish C samples from P samples are different when comparing ST with LT patients.

### All C and P samples, from both ST and LT patients, show a shared perturbed expression of a number of genes in comparison with healthy white matter

Even if C and P samples are obviously differentiated by the expression of many genes, one of the most important issues we wanted to address was to decipher the molecular signature, if any, shared by C and P samples when compared to normal controls of healthy white matter. To this aim, we compared our SAGE data from GBM patients to the RNA expression profile obtained through the same method on a healthy white matter sample. We found a limited number of genes (i.e. 11, Table 2a, *p* value < 0.05) underexpressed in both Cs and Ps of ST and LT patients, among which CNP and ENPP2 (encoding for 2′, 3′-cyclic nucleotide 3′ phosphodiesterase and for ectonucleotidepyrophosphatase/phosphodiesterase 2, respectively), both oligodendrocyte progenitor cell markers [20], and EDIL3, expressed by oligodendrocytes [20] and a marker of the “neural” subgroup of glioblastoma [13]. The overexpressed RNA molecules were slightly more (i.e. 21, Table 2b, *p* value < 0.05) than the underexpressed ones, and included microvascular, proliferation and survival markers of GBM, such as COL4A1 and TOP2A [21, 22], ECM molecules, as NID1 [23], markers of activated microglia, as CXCL14 [24], and RNAs characterizing the “classical” glioblastoma subtype, such as RGS12 and SOCS2 [13], also expressed in activated astrocytes [25], or even the “mesenchymal” subtype, such as TGFBI [13]. Notably, some of the overexpressed RNAs showed a comparable level of increase in both Cs and Ps compared to healthy white matter, likely indicating that some molecular events are taking place in the peritumor areas, which closely resemble those overtly occurring in frankly tumor regions. Several of these genes encode proteins playing their roles in the extracellular matrix (ECM), and/or in the regulation of cell-cell or cell-substrate adhesion, as collagen IV, CXCL14, and TGFBI. This indicates that ECM remodeling is a key process taking place in our samples.

**Table 2a:** List of 11 shared genes underexpressed in Cs and Ps, both LT and ST, *vs* healthy white matter

Underexpressed		Log_2_FCSTC	Log_2_FCSTP	Log_2_FCLTC	Log_2_FCLTP
**ADARB2**	RNA-specific adenosine deaminase B2	−4.110	−1.972	−4.043	−2.419
**C1ORF133**	chromosome 1 open reading frame 133	−2.786	−1.597	−3.521	−1.585
**CDC42EP1**	CDC42 effector protein (Rho GTPase binding) 1	−2.460	−1.541	−2.458	−1.873
**CNP**	2′, 3′-cyclic nucleotide 3′ phosphodiesterase	−3.876	−1.563	−4.801	−1.937
**EDIL3**	EGF-like repeats and discoidin I-like domains 3	−3.207	−1.158	−3.557	−1.602
**ELOVL1**	elongation of very long chain fatty acids (FEN1/Elo2, SUR4/Elo3, yeast)-like 1	−3.477	−1.227	−3.993	−1.640
**ENPP2**	ectonucleotide pyrophosphatase/phosphodiesterase 2	−4.128	−2.056	−5.594	−1.920
**ICOSLG**	inducible T-cell co-stimulator ligand	−2.048	−1.567	−3.516	−1.834
**PADI2**	peptidyl arginine deiminase, type II	−3.024	−1.471	−3.921	−1.937
**RPL21P44**	ribosomal protein L21 pseudogene 44	−1.521	−1.319	−1.875	−1.559
**TTC32**	tetratricopeptide repeat domain 32	−2.016	−1.581	−2.550	−1.799

The last four columns show the Log_2_FC of STC, STP, LTC and LTP, respectively, *vs* healthy white matter

**Table 2b:** List of the 21 shared genes overexpressed in Cs and Ps, both LT and ST, *vs* healthy white matter

Overexpressed		Log_2_FC vs ctr			
		STC	STP	LTC	LTP
ARC	activity-regulated cytoskeleton-associated protein	4.157	3.192	4.827	4.016
COL4A1	collagen, type IV, alpha 1	5.249	4.473	4.932	4.812
COL4A2	collagen, type IV, alpha 2	4.948	3.949	5.033	4.814
CXCL14	chemokine (C-X-C motif) ligand 14	4.467	2.880	4.110	4.104
EPHA4	EPH receptor A4	4.964	3.601	5.385	4.114
IDUA	iduronidase, alpha-L-	7.250	6.714	7.389	6.538
LMTK3	lemur tyrosine kinase 3	4.578	3.627	4.339	3.566
LOC100126784	hypothetical LOC100126784	3.687	2.720	3.282	2.817
LOC389831	hypothetical gene supported by AL713796	4.506	3.902	4.616	4.878
LPHN2	latrophilin 2	4.122	3.946	4.869	3.228
LRRC55	leucine rich repeat containing 55	2.891	2.839	2.780	3.005
LSM6	LSM6 homolog, U6 small nuclear RNA associated (S. cerevisiae)	5.128	4.788	5.323	5.102
METTL7B	methyltransferase like 7B	4.134	4.393	5.044	5.994
NID1	nidogen 1	3.646	2.987	3.540	2.685
PGM2L1	phosphoglucomutase 2-like 1	4.576	2.714	5.110	3.386
RGS12	regulator of G-protein signaling 12	4.506	3.871	4.941	4.695
SLCO2A1	solute carrier organic anion transporter family, member 2A1	5.916	5.189	5.598	5.205
SOCS2	suppressor of cytokine signaling 2	3.055	2.849	3.066	2.571
STAC2	SH3 and cysteine rich domain 2	6.034	3.353	5.606	3.102
TGFBI	transforming growth factor, beta-induced, 68 kDa	5.086	3.488	3.709	3.705
TOP2A	topoisomerase (DNA) II alpha 170 kDa	6.766	5.940	5.572	5.473

The last four columns show the Log_2_FC of STC, STP, LTC and LTP, respectively, *vs* healthy white matter

**Figure 3:**
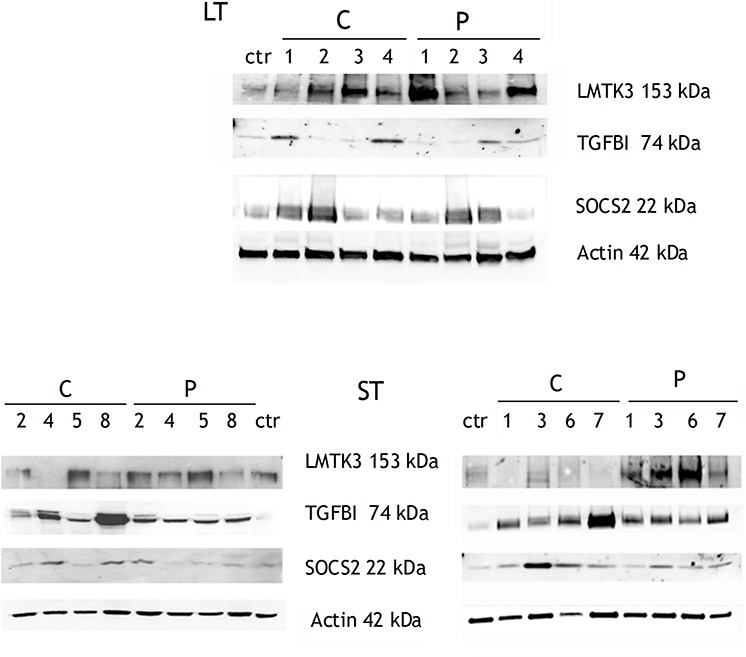
WB validation of three proteins whose mRNAs were overexpressed in C and P samples compared to healthy control The upper panel shows LT samples, whereas the lower panel shows ST samples. Among ST samples, ST2, ST4, ST5, ST8 (on the left) are the same samples already analyzed by SAGE; ST1, ST3, ST6, ST7 are additional ST samples.

We validated, by real-time qPCR, the differential expression of 8 mRNAs and 2 lncRNAs in in both STCs and STPs compared to healthy white matter, notably extending our SAGE results also to some new samples that had not been submitted to deep sequencing (Suppl. Table 5 and Suppl. Fig. 1). We then chose to validate by WB if the differential expression of some mRNAs was paralleled by a consistent modulation of the corresponding protein products too. In fact, we could confirm that LMTK3, encoding for Lemur Tyrosine Kinase 3 known to promote invasion in breast cancer [26], was widely overexpressed in both Cs and Ps of LT and ST patients (Figure 3). SOCS2 expression increase was confirmed at the protein level in the majority of tumor centers, while its overexpression in the peripheral areas was variable and in general less marked than in the centers (Fig. 3). Among the proteins whose increase we could validate, the strongest result was that of TGFBI, that we confirmed as overexpressed in nearly all LT, ST, P and C samples we assayed (Fig. 3).

### The differential expression of some key RNAs characterizes samples from short term and long term patients

A further key question that needs to be addressed in the comprehension of glioblastoma is the difference existing between the most frequent short survival patients (ST) and those that survive longer than 36 months after diagnosis, called long term survivors (LT). We thus searched our SAGE data for RNAs differentially expressed in these two categories, either in the comparison between tumor centers, or between peripheral areas. We started by focusing on RNAs whose expression difference between categories was statistically significant (i. e. FDR < 0.05). As shown in Table 3, 8 RNAs were overexpressed in STCs vs LTC, and only one underexpressed molecule reached this high statistical significance in the comparison. These RNAs include some whose involvement in GBM is soundly documented, such as PDGFRA [13], and also others, previously described in other types of solid tumors, but never in glioblastoma. Among these, two homeobox genes, HOXC10 and HOXD10, and TMSB15A, encoding for thymosin-beta-15A, a tumor motility gene promoting metastases in prostate cancer [27], all overexpressed in STCs vs LTCs. A strong difference in expression (Log_2_FC STC *vs* LTC = 4.4) was found for the mRNA CA3, encoding for carbonic anhydrase III, known to play an anti-oxidant role by working as an oxygen radical scavenger [28], and whose expression correlates with poor survival in GBM [29]. BCAR3, on the contrary, encoding for the SH2-containing signal transducer Breast cancer anti-estrogen resistance 3 [30], was clearly underexpressed (Log_2_FC = −2.07) in STCs vs LTCs. We chose to validate the differential expression of 3 out of such 8 molecules, and we could confirm all of them (Suppl. Fig. 2). A few genes were differentially expressed (FDR < 0.05) also between STPs and LTPs (Table 3), among which lumican (LUM), a proteoglycan with an established role in the control of tumor progression [31] that was underexpressed in STPs vs LTPs (Log_2_FC = −3.25).

**Table 3:** Differentially expressed (FDR < 0.05) genes between short-term survivors and long-term survivors tumor centers (upper panel), or between short-term survivors and long-term survivors peritumor areas (lower panel)

STC vs LTC				
NCBI Ref Seq	logFC	*P* Value	FDR	gene name
NM_005514	8, 459013	0, 000002	0, 003635	HLA-B
NM_017409	8, 069505	0, 000001	0, 001902	HOXC10
NM_002148	6, 016672	0, 000012	0, 010763	HOXD10
NR_002196	5, 114100	0, 000065	0, 026086	H19
NM_005181	4, 403243	0, 000003	0, 003707	CA3
NM_006206	4, 392766	0, 000148	0, 039570	PDGFRA
NM_021992	4, 074948	0, 000145	0, 039570	TMSB15A
NM_002196	3, 329623	0, 000018	0, 011555	INSM1
NM_003567	−2, 071359	0, 000231	0, 048284	BCAR3
**STP vs LTP**				
**NCBI Ref Seq**	**logFC**	***P* Value**	**FDR**	**gene name**
NM_005514	8, 295445	0, 000001	0, 001162	HLA-B
NM_030630	3, 089356	0, 000048	0, 017168	C17orf28
NM_002345	−3, 253966	0, 000045	0, 017158	LUM
NM_152679	−3, 369693	0, 000014	0, 006437	SLC10A4

When we extended our view of transcripts differentiating ST from LT samples also with a FDR higher than 0.05, among the RNAs more expressed in STCs vs LTCs, we found again TGFBI (Log_2_FC STC *vs* LTC = 1.8, *p* = 0.034), in agreement with its higher expression in all GBM samples *vs* healthy controls and likely as a sign of the greater aggressiveness of STCs vs LTCs. We also observed the overexpression of two molecules, COL6A2 (Log_2_FC STC vs LTC = 2.99, *p* = 0.0077) and the cell surface receptor CD44 (Log_2_FC STC *vs* LTC = 2.65, *p* = 0.019), commonly expressed in mesenchymal tissues [32] and associated with ECM remodeling [33]. Among the relevant genes overexpressed in STCs vs LTCs, we also detected two key transcription factors, SOX4 and SOX11 (Log_2_FC STC vs LTC = 2.58, *p* = 0.000458, and 2.75, *p* = 0.00236, respectively), usually associated with neural stem cells [34]. In particular SOX4, a direct TGF-β target gene, was demonstrated to sustain tumorigenicity of glioma-initiating cells [35]. Interestingly, when we compared the peripheral areas of ST *vs* LT patients, we found the overexpression (Log_2_FC STP vs LTP = 1.6, *p* = 0.0178) of IGFBP5, encoding for insulin-like growth factor binding protein 5, previously shown to be expressed by activated astrocytes in retinoblastoma, where they cooperate with tumor cells to promote tumorigenesis [36].

We then submitted our differentially expressed RNAs to a category enrichment analysis by using DAVID bioinformatics resources [37, 38], to understand if ST tumors are distinguished from LT ones by specific cellular components or molecular pathways. As shown in Table 4, when considering the RNAs overexpressed in STCs *vs* LTCs, the extracellular matrix is the cellular component clearly prevalent, whereas the categories highly enriched in RNAs underexpressed in STCs vs LTCs are those of “synapse”, “cell junction”, “cation channel complex”, and similar ones related to neuronal development and differentiation. Also the molecular pathways enriched in STCs or LTCs are clearly different (Table 5): genes overexpressed in STCs *vs* LTCs are enriched in KEGG pathways such as “cell cycle”, and, significantly, “TGF-beta signaling pathway”. On the contrary, and in agreement with the cellular component analysis, genes underexpressed in STCs vs LTCs are enriched in pathways involving the function of several types of synapses, calcium signaling pathway, and also chemokine signaling pathway.

**Table 4:** GO Cellular Components (CC) categories enrichment for genes either overexpressed (A) or underexpressed (B) in STCs vs LTCs

A					
Id category	Name category	Adjusted *p* value	Non corr *p* value	Fold enrichment	# Genes
GO:0005576	extracellular region	2.85e-07	4.57e-10	2.52	48
GO:0044421	extracellular region part	2.85e-07	6.95e-10	3.32	33
GO:0005615	extracellular space	2.99e-06	1.10e-08	3.50	27
GO:0031012	extracellular matrix	2.91e-04	1.42e-06	4.19	16
GO:0005578	proteinaceous extracellular matrix	1.78e-02	1.09e-04	3.68	12
GO:0031941	filamentous actin	1.10e-01	8.08e-04	16.15	3
GO:0009897	external side of plasma membrane	1.79e-01	1.75e-03	4.06	7
GO:0031091	platelet alpha granule	1.79e-01	1.65e-03	7.89	4
GO:0000808	origin recognition complex	2.01e-01	2.45e-03	26.31	2
GO:0005664	nuclear origin of replication recognition complex	2.01e-01	2.45e-03	26.31	2
GO:0044433	cytoplasmic vesicle part	2.20e-01	2.95e-03	2.66	11
GO:0005796	Golgi lumen	2.67e-01	3.91e-03	6.23	4
GO:0031093	platelet alpha granule lumen	4.60e-01	7.29e-03	7.56	3
GO:0034774	secretory granule lumen	5.06e-01	8.66e-03	7.10	3

B					
Id category	Name category	Adjusted *p* value	Non corr *p* value	Fold enrichment	# Genes
GO:0045202	synapse	1.49e-11	1.13e-14	4.82	35
GO:0030054	cell junction	9.96e-09	1.52e-11	3.43	39
GO:0044456	synapse part	8.50e-07	1.94e-09	4.32	24
GO:0016020	membrane	2.72e-05	9.72e-08	1.34	165
GO:0043005	neuron projection	2.72e-05	1.03e-07	3.04	29
GO:0071944	cell periphery	3.51e-04	1.60e-06	1.50	104
GO:0097060	synaptic membrane	9.46e-04	5.04e-06	4.32	14
GO:0045211	postsynaptic membrane	9.49e-04	5.78e-06	4.58	13
GO:0005886	plasma membrane	1.02e-03	6.95e-06	1.48	100
GO:0032591	dendritic spine membrane	1.74e-03	1.32e-05	49.97	3
GO:0042995	cell projection	1.78e-03	1.57e-05	2.07	38
GO:0034702	ion channel complex	1.78e-03	1.62e-05	4.16	13
GO:0044425	membrane part	1.91e-03	1.89e-05	1.34	131
GO:0034703	cation channel complex	2.87e-03	3.06e-05	5.05	10
GO:0043025	neuronal cell body	1.06e-02	1.21e-04	3.24	14
GO:0031224	intrinsic to membrane	1.44e-02	1.75e-04	1.32	115
GO:0030425	dendrite	1.85e-02	2.45e-04	2.89	15
GO:0008021	synaptic vesicle	1.85e-02	2.53e-04	4.85	8
GO:0044297	cell body	2.32e-02	3.36e-04	2.93	14
GO:0031226	intrinsic to plasma membrane	3.53e-02	5.37e-04	1.76	37
GO:0005887	integral to plasma membrane	3.70e-02	5.91e-04	1.77	36
GO:0016021	integral to membrane	4.10e-02	6.87e-04	1.29	110
GO:0043204	perikaryon	4.23e-02	7.41e-04	6.94	5
GO:0032589	neuron projection membrane	4.61e-02	8.76e-04	9.19	4
GO:0008328	ionotropic glutamate receptor complex	4.61e-02	8.76e-04	9.19	4
GO:0012505	endomembrane system	5.89e-02	1.17e-03	1.58	46
GO:0070044	synaptobrevin 2-SNAP-25-syntaxin-1a complex	6.42e-02	1.32e-03	33.32	2
GO:0044448	cell cortex part	1.45e-01	3.08e-03	4.21	6
GO:0034704	calcium channel complex	1.47e-01	3.25e-03	6.50	4
GO:0034705	potassium channel complex	1.70e-01	4.01e-03	4.76	5
GO:0008076	voltage-gated potassium channel complex	1.70e-01	4.01e-03	4.76	5
GO:0031045	dense core granule	1.79e-01	4.48e-03	19.04	2
GO:0044459	plasma membrane part	1.79e-01	4.47e-03	1.47	47
GO:0014701	junctional sarcoplasmic reticulum membrane	2.64e-01	7.54e-03	14.81	2
GO:0016529	sarcoplasmic reticulum	2.64e-01	7.63e-03	5.13	4
GO:0000145	exocyst	2.64e-01	7.54e-03	14.81	2
GO:0030136	clathrin-coated vesicle	2.64e-01	6.89e-03	2.51	10
GO:0030672	synaptic vesicle membrane	2.64e-01	7.13e-03	5.23	4
GO:0031201	SNARE complex	2.80e-01	8.30e-03	7.14	3
GO:0030424	axon	3.04e-01	9.27e-03	2.41	10

The grey background highlights GOCCs with an adjusted *p*-value < 0.05. Adjusted *p*-value represents the Benjamini – corrected *p*-value

**Table 5:** KEGG categories enrichment for genes either overexpressed (A) or underexpressed (B) in STCs vs LTCs

A					
Id category	Name category	Adjusted *p* value	Non corr *p* value	Fold enrichment	# Genes
hsa05219	Bladder cancer	4.12e-02	3.30e-04	11.88	4
hsa04115	p53 signaling pathway	4.12e-02	1.74e-04	9.39	5
hsa04110	Cell cycle	4.12e-02	4.15e-04	6.08	6
hsa05144	Malaria	7.75e-02	1.04e-03	8.81	4
hsa05134	Legionellosis	1.55e-01	2.70e-03	6.81	4
hsa05202	Transcriptional misregulation in cancer	1.55e-01	3.12e-03	4.12	6
hsa04630	Jak-STAT signaling pathway	3.31e-01	7.77e-03	4.02	5
hsa04512	ECM-receptor interaction	3.67e-01	9.85e-03	4.73	4
hsa05214	Glioma	4.19e-01	1.41e-02	5.90	3
hsa04621	NOD-like receptor signaling pathway	4.19e-01	1.41e-02	5.90	3
hsa05218	Melanoma	4.82e-01	1.78e-02	5.40	3
hsa04060	Cytokine-cytokine receptor interaction	6.17e-01	2.49e-02	2.64	6
hsa04610	Complement and coagulation cascades	6.54e-01	2.85e-02	4.51	3
hsa05132	Salmonella infection	6.83e-01	3.21e-02	4.31	3
hsa04350	TGF-beta signaling pathway	7.13e-01	3.59e-02	4.12	3

B					
Id category	Name category	Adjusted *p* value	Non corr *p* value	Fold enrichment	# Genes
hsa05032	Morphine addiction	1.15e-04	2.47e-07	7.30	11
hsa04020	Calcium signaling pathway	1.34e-04	5.77e-07	5.03	14
hsa04727	GABAergic synapse	2.30e-04	1.49e-06	6.92	10
hsa04723	Retrograde endocannabinoid signaling	3.33e-04	2.88e-06	6.44	10
hsa04970	Salivary secretion	9.57e-04	1.03e-05	6.36	9
hsa04724	Glutamatergic synapse	1.38e-03	1.79e-05	5.26	10
hsa04726	Serotonergic synapse	6.02e-03	1.03e-04	4.77	9
hsa05414	Dilated cardiomyopathy	6.02e-03	1.04e-04	5.42	8
hsa04080	Neuroactive ligand-receptor interaction	1.49e-02	2.89e-04	3.06	13
hsa04666	Fc gamma R-mediated phagocytosis	3.19e-02	6.88e-04	4.69	7
hsa04070	Phosphatidylinositol signaling system	5.85e-02	1.39e-03	4.87	6
hsa04144	Endocytosis	6.14e-02	1.73e-03	2.81	11
hsa04270	Vascular smooth muscle contraction	6.14e-02	1.86e-03	3.96	7
hsa04725	Cholinergic synapse	6.14e-02	1.68e-03	4.03	7
hsa05410	Hypertrophic cardiomyopathy (HCM)	7.37e-02	2.38e-03	4.38	6
hsa05033	Nicotine addiction	7.58e-02	2.77e-03	6.74	4
hsa00604	Glycosphingolipid biosynthesis	7.58e-02	2.78e-03	10.37	3
hsa05214	Glioma	7.65e-02	2.97e-03	5.05	5
hsa04540	Gap junction	9.38e-02	3.85e-03	3.98	6
hsa04010	MAPK signaling pathway	9.38e-02	4.66e-03	2.48	11
hsa04721	Synaptic vesicle cycle	9.38e-02	4.63e-03	4.56	5
hsa04720	Long-term potentiation	9.38e-02	4.10e-03	4.69	5
hsa04728	Dopaminergic synapse	9.38e-02	4.53e-03	3.38	7
hsa05412	Arrhythmogenic right ventricular cardiomyopathy (ARVC)	9.65e-02	5.21e-03	4.44	5
hsa05031	Amphetamine addiction	9.65e-02	5.21e-03	4.44	5
hsa04260	Cardiac muscle contraction	1.04e-01	5.83e-03	4.32	5
hsa04062	Chemokine signaling pathway	1.63e-01	9.48e-03	2.70	8

The grey background highlights KEGG categories with an adjusted *p*-value < 0.05. Adjusted *p*-value represents the Benjamini – corrected *p*-value

Altogether, these results indicate that ST tissues differ from LT ones, especially for the expression of markers of TGF-β pathway, of reactive astrocytes, extracellular matrix remodeling and tumor cell motility.

### The miRNome profiling of GBM centers and peritumor areas reveals microRNA molecules differentially expressed in all tumor centers vs their own peritumor areas

**Figure 4:**
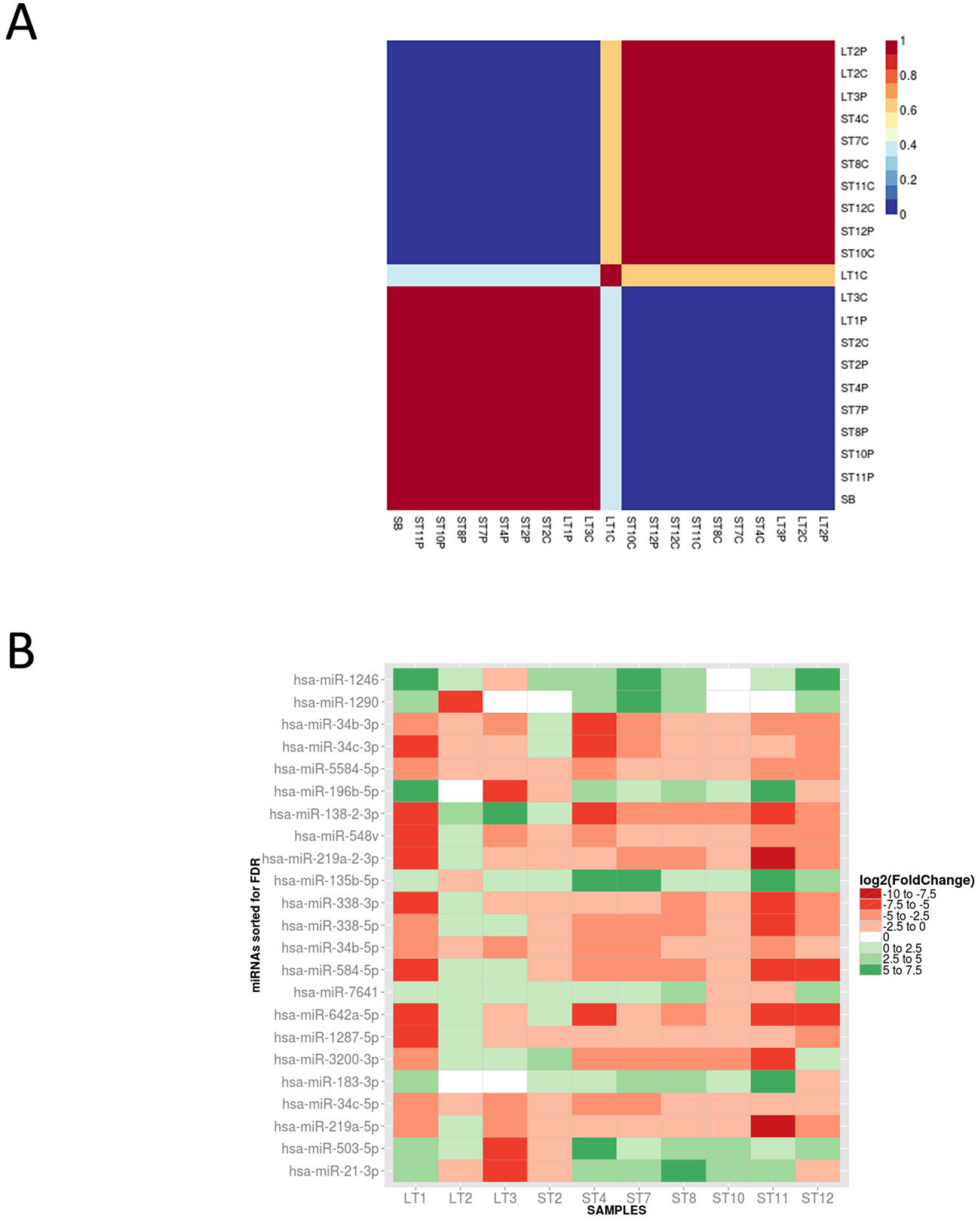
A. MicroRNA expression can correctly clusterize GBM C and P samples Consensus non-negative matrix factorization clustering of miRNA expression data distinguishes two clusters: one is prevalently made of P samples and includes the healthy control (SB), the other is enriched by C samples. **B.** A selected number of miRNAs can clearly distinguish each C sample from its own P region. Shown is the fold change heatmap of differentially expressed miRNAs in the paired comparison P/C cell types. Each box refers to a specific C/P cell type comparison, the color scale reflects the relative Log2(Fold Change) (green indicates over expression in C samples, red indicates underexpression).

In order to improve our molecular picture of glioblastoma tissues, we performed a microRNA deep sequencing analysis on a cohort of samples almost completely overlapping (3 out of 4 LT and 7 out of 9 ST) with those already studied by SAGE. The data we obtained were initially employed to check whether the sole miRNome expression is able, *per se*, to separate our samples into clinically relevant classes. In fact, as shown in Figure 4a, total miRNA expression could correctly clusterize most of GBM C and P samples, and, as expected, placed the healthy white matter control at one edge of the cluster prevalently made by P samples, opposite to C tissues. We then asked the same question previously posed with the SAGE data, i.e. which microRNAs are differentially expressed in all C/P pairs. For this purpose we made a paired analysis comparing the expression level of each C and P sample in the 10 analyzed samples. The heatmap shown in Figure 4b represents the miRNAs with expression significantly different (FDR < 0.05) in this comparison. Among miRNAs overexpressed in C vs P samples, we found miR-21-3p, miR-196b-5p, miR-135b-5p, miR-183-3p, known as “oncomiRs” in several tumors, including glioblastoma [39–42]. In addition, we found a few other overexpressed miRNAs, such as miR-1246, miR-1290, miR-7641, and miR-503-5p, whose role in cancer or in glioblastoma hasn't been investigated yet. We also found a larger number of underexpressed miRNAs in C vs P samples, among which miR-219a and miR-338-3p and miR-338-5p, with an established role in oligodendrocyte maturation [43], miR-34b and miR-34c, widely recognized as tumor suppressor miRNAs in general and specifically in GBM [44], but also several others, whose role in cancer is not known. We further confirmed our findings by validating also by real time qPCR the expression of 5 microRNAs among those shown in Figure 4b, and of three other randomly chosen ones (Suppl. Fig. 3).

### A common set of microRNAs distinguishes C and P samples from healthy white matter

As for the SAGE analysis, we were interested in the comparison of C and P samples *vs* the healthy control, in search of molecular signatures able to differentiate not only frankly tumoral regions, but also the peritumoral areas, and thus corroborating the involvement of such regions in tumor onset, growth and support. We selected those miRNAs with a Log_2_FC *vs* healthy control of at least 1.5 for overexpressed miRNAs, or with a Log_2_FC vs healthy control of at least −1.5 for the underexpressed ones. Among overexpressed miRNAs, listed in Table 6a, we report several known “oncomirs”, such as miR-10b, miRNAs of the miR-17-92 cluster, miR-21, miR-148 and others, largely described for their roles in cancer and in glioblastoma specifically [45–47], even if not described in the peritumoral areas. In addition, we found a set of miRNAs, among which miR-503-5p, not only overexpressed in C and P samples vs healthy white matter, but also in Cs *vs* Ps (see Figure 4b), suggesting a direct correlation of its expression with the presence of tumor cells. Moreover, the average expression of two miRNAs, miR-18a-5p and miR-503-5p, resulted higher in ST tumors as compared to LT ones (Log_2_FC STC *vs* LTC = 2.52, and 2.25, respectively), suggesting a correlation between their expression and tumor aggressiveness. In this class of microRNAs correlated with the glioblastoma state, we inserted also three miRNAs that, apart from distinguishing P and C samples from healthy controls, showed also a differential expression between those peripheral areas with a detectable tumor cell infiltration and the non-infiltrated ones. These three miRNAs, miR-182-5p, miR-183-5p and miR-96-5p (Log_2_FC PNI vs PI = −3.96, −3.76, −3.79, respectively, with respective *p*-values of 8.96 × 10^−5^, 1.7 × 10^−4^, 1.64 × 10^−4^), interestingly belong to the same genomic cluster located on chromosome 7, and own a recognized role as mediators of TGFβ signaling in glioblastoma [48]. In the comparisons with healthy controls too, as for the C/P comparisons, we found a larger number of underexpressed microRNAs in tumor samples (both P and C, LT and ST) compared to healthy control (Table 6c). Among these, several miRNAs previously described as downregulated in glioblastoma were present, such as miR-128, miR-124, miR-129 [49, 50], but also many others, not yet described in this context, were found. Moreover, only four miRNAs, miR-146b-3p, miR-483-3p, miR-184, and miR-187-3p, were overexpressed in non infiltrated peritumoral areas *vs* infiltrated ones (Log_2_FC PNI vs PI = 3.17, *p* = 1.84 × 10^−4^, 3.18, *p* = 1.81 × 10^−4^, 3.66, *p* = 2.03 × 10^−5^, 3.83, *p* = 9.43 × 10^−6^, respectively).

**Table 6a:** List of microRNAs overexpressed in C and P samples *vs* healthy white matter (HC)

miRNA	HC	LTC	LTP	STC	STP
hsa-miR-106b-5p	119	537	1025	2887	1222
hsa-miR-10b-5p	11	557	1445	1589	311
hsa-miR-1248	18	255	128	174	101
hsa-miR-1260a	151	1033	906	933	431
hsa-miR-1260b	14	101	128	66	82
hsa-miR-148a-3p	89	529	266	588	300
hsa-miR-16-2-3p	15	51	72	156	50
hsa-miR-182-5p	11	75	52	1322	230
hsa-miR-183-5p	2	51	15	508	87
hsa-miR-18a-5p	35	135	441	460	329
hsa-miR-210-3p	3	214	212	355	199
hsa-miR-21-3p	0	37	37	68	10
hsa-miR-21-5p	1132	15222	40876	51388	7627
hsa-miR-24-2-5p	6	59	76	70	36
hsa-miR-3065-5p	7	134	47	36	32
hsa-miR-454-3p	33	208	173	344	319
hsa-miR-503-5p	5	23	22	74	17
hsa-miR-92b-5p	2	33	108	74	18
hsa-miR-93-5p	1439	6443	7835	12378	6080

The numbers shown represent the cpm median values for each class of samples. Only miRNAs with a module fold change (log2) higher than 1.5 in all four comparisons (LTC vs HS, LTP vs HS, STC vs HS, STP vs HS) and with at least 50 cpm in one of the 5 samples are reported

In order to investigate which functions can be most likely affected by the modulated miRNAs, we performed a DIANA miRPath analysis [51], that identifies KEGG pathways involving the mRNAs predicted to be targeted by the modulated miRNAs. The highest ranking pathways predicted to be affected by either overexpressed or underexpressed miRNAs are listed in Table 6b and 6d, respectively: many pathways are expected to be influenced by both classes of miRNAs, while some others are preferentially related to one class. For example, overexpressed miRNAs are predicted to affect “Regulation of actin cytoskeleton” and “p53 signaling” pathways, clearly representing the morphological modifications typical of tumor cells, and the complex molecular interactions downstream of p53.

**Table 6b:** KEGG pathways enriched for mRNAs predicted to be targeted by miRNAs listed in Table 6a

KEGG PATHWAY	*P* value	# genes	# miRNAs
Neurotrophin signaling pathway	5.91e-19	54	18
Regulation of actin cytoskeleton	1.82e-18	84	16
PI3K-Akt signaling pathway	1.82e-18	118	17
Axon guidance	2.78e-17	57	16
TGF-beta signaling pathway	2.76e-15	37	16
ErbB signaling pathway	8.16e-13	40	15
Endocytosis	1.08e-11	71	17
Ubiquitin mediated proteolysis	7.57e-12	53	16
Focal adhesion	1.44e-11	71	16
Pathways in cancer	3.91e-11	114	18
p53 signaling pathway	6.66e-11	30	14
MAPK signaling pathway	6.66e-11	87	17
Wnt signaling pathway	1.02e-10	58	18

The number of genes targeted in each pathway and the number of miRNAs predicted to target those mRNAs are indicated in the third and fourth column, respectively

**Table 6c:** List of microRNAs underexpressed in C and P samples *vs* healthy white matter (HC)

miRNA	HC	LTC	LTP	STC	STP
hsa-miR-1185-5p	59	16	8	10	10
hsa-miR-1224-3p	245	29	7	4	25
hsa-miR-124-5p	359	83	13	36	26
hsa-miR-1249	1468	153	199	64	275
hsa-miR-127-5p	104	23	7	23	11
hsa-miR-128-3p	28670	4382	2616	3413	6134
hsa-miR-129-1-3p	343	55	16	16	61
hsa-miR-129-2-3p	6142	1786	321	292	695
hsa-miR-129-5p	494	126	60	29	94
hsa-miR-137	446	98	18	41	100
hsa-miR-139-3p	74	20	0	0	6
hsa-miR-139-5p	20625	3441	1018	547	2306
hsa-miR-154-3p	216	43	35	63	71
hsa-miR-3200-3p	416	19	5	0	14
hsa-miR-323a-3p	2795	610	246	289	389
hsa-miR-323b-3p	330	68	24	34	63
hsa-miR-326	1204	423	216	103	183
hsa-miR-329-3p	1241	255	53	125	236
hsa-miR-369-3p	142	38	15	47	30
hsa-miR-376a-5p	100	32	6	19	16
hsa-miR-381-5p	89	27	3	8	11
hsa-miR-431-3p	81	16	0	3	4
hsa-miR-485-3p	387	90	16	41	38
hsa-miR-487a-3p	226	57	29	51	55
hsa-miR-487b-3p	5864	1661	414	412	773
hsa-miR-491-5p	166	54	8	4	12
hsa-miR-504-5p	460	107	58	9	16
hsa-miR-582-5p	331	46	54	85	75
hsa-miR-628-5p	504	163	152	44	132
hsa-miR-6511b-3p	190	50	46	25	47
hsa-miR-656-3p	123	23	11	14	26
hsa-miR-668-3p	57	12	3	4	5
hsa-miR-7-1-3p	521	150	148	146	179
hsa-miR-7-2-3p	143	19	10	12	22
hsa-miR-769-3p	271	42	26	23	27
hsa-miR-769-5p	355	60	19	79	104
hsa-miR-770-5p	190	55	4	8	12
hsa-miR-874-3p	3834	1002	590	470	748
hsa-miR-99b-5p	1186	71	217	121	171

The numbers shown represent the cpm median values for each class of samples. Only miRNAs with a module fold change (log2) higher than 1.5 in all four comparisons (LTC vs HS, LTP vs HS, STC vs HS, STP vs HS) and with at least 50 cpm in one of the 5 samples are reported

**Table 6d:** KEGG pathways enriched for mRNAs predicted to be targeted by miRNAs listed in Table 6c

KEGG PATHWAY	*P* value	# genes	# miRNAs
MAPK signaling pathway	1.37e-41	135	34
PI3K-Akt signaling pathway	1.43e-40	168	33
Focal adhesion	3.05e-31	104	31
Wnt signaling pathway	5.92e-29	89	28
Axon guidance	6.27e-27	78	33
Pathways in cancer	4.06e-25	170	34
Dopaminergic synapse	6.08e-25	78	35
Ubiquitin mediated proteolysis	8.91e-24	77	31
Neurotrophin signaling pathway	2.71e-22	72	32
Glioma	5.01e-20	44	25
ErbB signaling pathway	1.61e-18	57	31
TGF-beta signaling pathway	1.94e-18	51	27

The number of genes targeted in each pathway and the number of miRNAs predicted to target those mRNAs are indicated in the third and fourth column, respectively. The significance level is indicated by a corrected *p*-value

### C samples show a reduced percentage of A to G editing of miR-376c-3p

**Figure 5:**
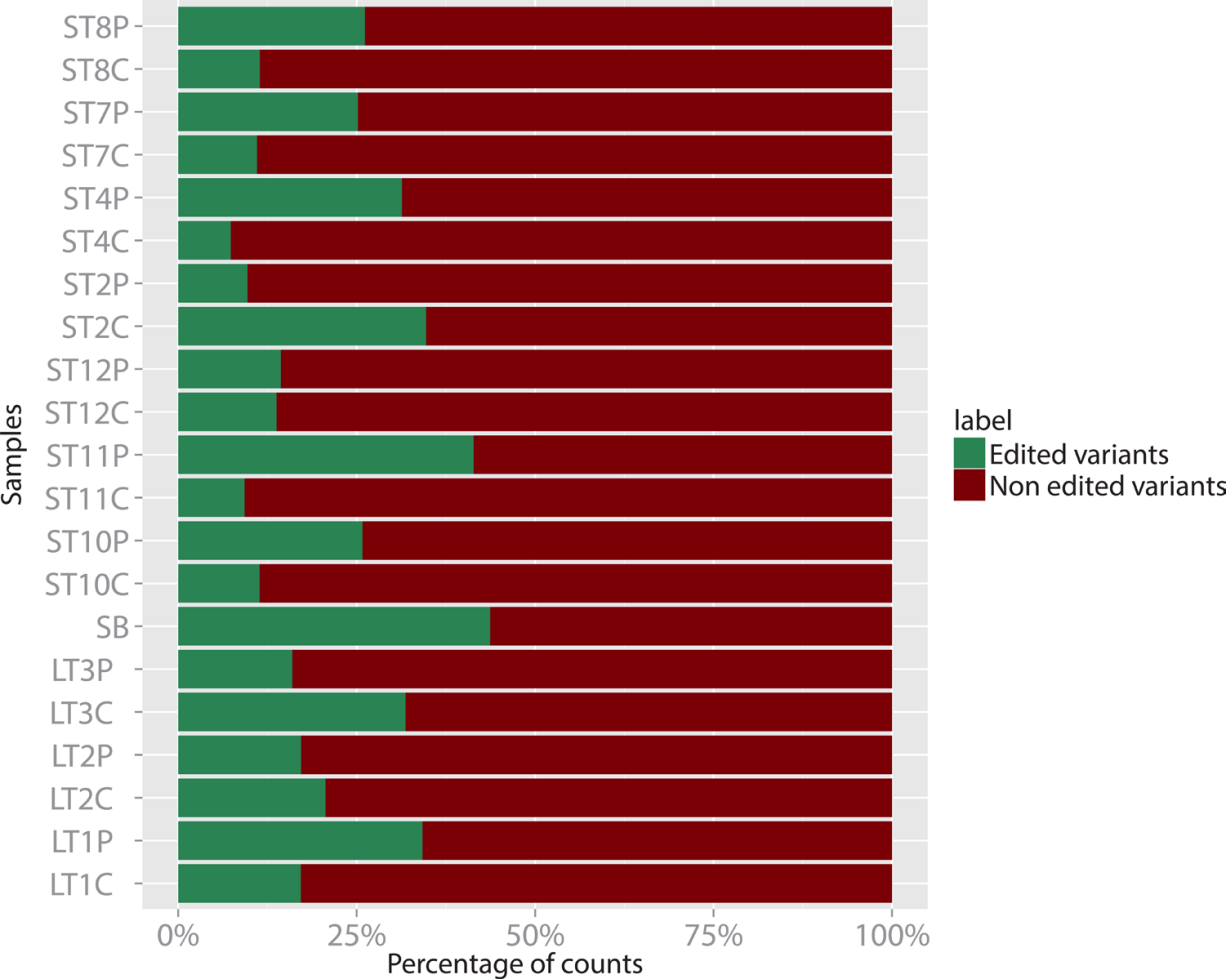
Percent distribution of edited (in green) vs non edited (in purple) variants of miR-376c-3p in 7 C/P pairs of ST samples, 3 C/P pairs of LT samples, and in one healthy white matter sample (SB)

The editing of RNA sequences by adenosine deaminases is an important mode of modulation of RNA function [52], and has been shown to affect microRNA sequences too, with several functional consequences, among which the change in targeted mRNAs [53]. We then studied our microRNA sequencing data in search of possible sequence isoforms that may originate from an A to G editing, and we found that miR-376c-3p, even if not differentially expressed in C *vs* P samples or in tumor samples *vs* healthy control, was less edited in C samples with respect to the P ones, that owned a fraction of edited variants comparable to that of the healthy white matter. Specifically, the edited forms harbored a G in place of an A at nucleotide 6 starting from the 5′ of the mature miRNA, thus affecting its “seed” sequence and potentially its target mRNAs. As shown in Figure 5, most P samples showed a relative amount of edited miR-376c-3p which was comparable to that of the healthy control sample, while the majority of C samples owned a reduced percentage of the edited forms in favor of the unedited ones.

In order to verify the significance of these observation, we performed a chi-squared test by considering two independent groups, one made by the C samples group and the other made by the P samples counterpart. For each group, we summed all the normalized counts of the edited variants of the miRNA 376c-3p, separating them from the unedited ones. The two sample test for equality of proportion with continuity correction gave a *p*-value = 3.6 × 10^−12^ (X-squared of 48.3). Interestingly, we also noticed that LTC samples display, on average, a fraction of edited variants higher than the STC samples. For these two groups too, we obtained a *p*-value = 2.2 × 10^−16^ (X-squared of 245.4).

To understand if the editing of miR-376c-3p might affect its mRNA target cohort, we performed a target prediction analysis of the two forms, edited *vs* unedited, and then submitted the two lists of predicted mRNAs to a functional annotation clustering analysis. We thus found that the edited forms are predicted to target mRNAs grouping in clusters such as “Hedgehog signaling pathway” and “Wnt signaling pathway” (Suppl. Table 2a), typically characterizing tumors and GBM in particular. On the contrary, unedited miR-376c-3p predicted targets (Suppl. Table 2b) are enriched in mRNAs coding for proteins involved in sugar transmembrane transport and in apoptosis, completely absent among those predicted to be targets of edited miR-376c-3p. Of note, among the predicted targets of unedited miR-376c-3p, overexpressed in C samples, we found key cell control genes, such as CDKN1A (coding for p21) or the microRNA processing enzyme Drosha.

All these data indicate that a certain loss of the physiological A to G editing of miR-376c-3p occurs in GBM samples compared to healthy tissues and also to the peripheral areas, and that this likely induces a shift in the mRNAs targeted by miR-376c-3p, in turn driving GBM cells towards cell proliferation and other typically tumoral functions.

## DISCUSSION

We undertook a multifaceted molecular analysis of a group of tissue samples from glioblastoma patients, and specifically focused on two main issues: i) RNAs whose expression is perturbed not only in glioblastoma tumor samples (Cs), but also in the peritumoral areas (Ps), compared to white matter healthy controls; ii) molecules distinguishing the tissues from “long term” (LT) GBM patients (that survived longer than 36 months after diagnosis) from those from the most common “short term” (ST) ones. The rationale behind these two main goals of our work is the idea that the different survival of LT compared to ST patients might be explained by different molecular signatures, to which a significant contribute may be provided by the peritumoral areas, including multiple cell types. More generally, our view, supported by the data we are presenting, is that peritumoral areas are actively involved in glioblastoma even when a clear tumor phenotype is not detectable.

### A mesenchymal signature, indicative of a possible infiltration of stromal cells, characterizes C and P samples compared to healthy white matter and specifically short-term tumors

The analysis of modulated RNAs affecting both tumor centers and peritumoral areas unveiled the overexpression of several molecules belonging to the “mesenchymal signature” of glioblastoma [54, 13, 10], that greatly overlaps with the infiltration of tumor-supporting “stromal cells”, a role that in brain is played by reactive astrocytes and microglia.

Among these RNAs, in our view the most notable ones are CXCL14 and TGFBI. We find CXCL14 overexpressed in C and P samples (both ST and LT) *vs* healthy white matter; however, while its expression levels are comparable in LTCs and in LTPs, it is overexpressed in the majority (7/9) of STC *vs* STP samples. Indeed, we confirmed CXCL14 expression by IHC (Suppl. Fig. 4) in the cytosol and extracellular region of reactive astrocytes in STC samples, whereas STP samples showed a lighter staining in the cytoplasm of some astrocytes, likely representing the activated ones. A few works have been published using only murine models, showing that CXCL14 is expressed by activated microglia after induction by GBM conditioned medium [24]; our finding of CXCL14 increased expression in STCs compared to STPs may reflect a higher level of microglial or more probably reactive astrocyte infiltration in ST tumor centers compared to peritumor areas, which, on the contrary, doesn't characterize LT tumor centers when compared to their own peritumor areas. This hypothesis could be supported by our data collected in the comparison of STCs vs STPs: among the RNAs overexpressed in STCs *vs* STPs, COL1A1, COL1A2 and COL5A1 are known markers of the mesenchymal glioblastoma subtype, as well as IGFBP6, DCBLD2 and OLFM1, all expressed by microglia or reactive astrocytes [13, 10]. Notably, such an enrichment in genes expressed by stromal cells is absent among RNAs overexpressed in LTCs vs LTPs.

Another relevant example is TGFBI which, besides its recognized role as a marker of the mesenchymal class of glioblastoma [13], was described, in conjunction with SOX4, as a mediator of non-SMAD mediated TGF-beta signaling pathways in glioblastoma [55]. TGFBI is mainly secreted in the extracellular matrix, where it interacts with several other components, such as collagens, among which collagen IV alpha 2, whose mRNA is among those overexpressed in all C and P samples of our study. Our data thus not only confirm those reported by Lin and coworkers [55], but extend the significance of TGFBI overexpression also to peritumoral regions. Notably, when we searched for TGFBI protein expression by IHC, we found it in the extracellular space and in the basement membrane of vessels of C tissues, while it marked less intensely single cells in P samples (Suppl. Fig. 4), with a difference of expression reflecting that observed for TGFBI mRNA by SAGE. In P regions showing clear evidence of tumor cell infiltration, TGFBI staining was found extracellularly and in the basement membrane of vessels, reflecting the staining observed in C samples (not shown). Specific studies addressing the question of which cell types produce and secrete TGFBI in GBM microenvironment are surely needed, in order to understand its possible paracrine or autocrine roles in this context.

In general, we observed that, among overexpressed mRNAs shared throughout all C and P samples, the level of modulation (Log_2_FC, see Table 2b) *vs* healthy white matter was frequently comparable in LTCs and LTPs, while it was almost always higher in STCs compared to STPs. We propose that this may be explained by an enhanced transformation stage characterizing STCs, but at the same time may imply that several molecular pathways supporting tumor growth are already “switched on” in peritumor areas.

### An “oncomir” expression pattern distinctive of TGFβ activation characterizes all C and P samples compared to healthy white matter

Also when we analyzed the microRNAs extensively overexpressed throughout all C and P samples compared to healthy white matter, we found many that may represent both signs of TGFβ active pathways and mediators of a general state of immune escape typically sustaining glioma growth. In fact, among the several known “oncomiRs” overexpressed in our samples, we found miR-106b and miR-93, recently shown to target NKG2DL, a ligand of the activating receptor of natural killer (NK) cells NKG2D, and thus suggested to contribute to the immune evasion typical of glioblastoma cells [56]. Mir-183, that we found to characterize most of our samples and also to distinguish the infiltrated *vs* the non-infiltrated peritumoral areas, is a TGFβ-induced miRNA previously reported to suppress tumor-associated natural killer cells, thus explaining one of the ways through which TGFβ plays its immunosuppressive roles in tumor microenvironment [57]. We can recognize several further microRNAs positively correlated with TGFβ role as a supporter of tumor growth: miR-21, shown to be induced by TGFβ1 and to target SMAD7, thus leading to the TGFβ-promoted formation of cancer associated fibroblasts [58]. Mir-106 too was described as deeply involved in TGFβ protumorigenic signaling through targeting of SMAD7, in turn inducing EMT in breast cancer [59], similarly to miR-10b, induced by TGFβ1 and promoting EMT in breast cancer [60]. Specifically in glioblastoma, miR-182 is induced by TGF-β, leading to prolonged NF-κB activation in a glioma subset [48]. Even among the underexpressed miRNAs we found possible indications of TGFβ action, such as the reduced expression of miR-127, demonstrated to be under the negative control of TGFβ in hepatocellular carcinoma [61]. To our knowledge, ours is the first work where a systematic measurement of microRNA expression is performed in glioblastoma peritumoral areas, and the results yielded by such a study, together with the gene expression analysis of the same samples, indicate that peritumoral regions share with frankly tumor areas a mRNA and microRNA signature distinctive of TGFβ activation and possibly of the involvement of cell types mediating a general immunosuppressive condition.

### ST tumor and peritumor areas, compared to LT ones, show an enrichment of mRNAs known to be expressed by activated astrocytes and microglia

When we compared tumor centers to peritumoral areas, in the ST group we observed several signs of a higher participation of reactive cell types, such as microglia and reactive astrocytes, that could be depicted also as a generally more “mesenchymal” feature compared to what we found in LTs. Suggestive of a such a view, in the STC vs STP comparison, but not in the LTC-LTP one, we found the overexpression of prolylendopeptidase, a serine protease digesting ECM and typically expressed by microglia [62], and that of ARHGAP29, expressed by reactive astrocytes and microglia [10]. An even stronger indication was provided by the direct comparison of STCs vs LTCs, that underscored a clear enrichment of RNAs known to be expressed by activated astrocytes and microglia, such as carbonic anhydrase III (CA3), CD44 and TGFBI. In particular, CD44 is the receptor for hyaluronate and osteopontin [63, 64], critical for cell adhesion and invasion, and is a key marker of reactive astrocytes, whose infiltration in the tumor and in the peritumoral area correlates with a worse prognosis [11]. It has also been recently shown that CD44 expression, being a marker of TNFalpha/NFkB-induced mesenchymal differentiation of glioblastoma, correlates with poor radiation response and shorter survival [54]. Our IHC results show CD44 localized at the membranes of specific cells, most likely reactive astrocytes, in C samples, and throughout the intricate net of cell processes in P tissues (Suppl. Fig. 4). A gene that resulted strongly underexpressed in our STC samples *vs* the LTC ones was BCAR3, whose role in glioblastoma has never been revealed before. However, it has very recently reported that it is downregulated by TGFβ and it can also inhibit TGFβ/SMAD signaling in breast cancer, where its expression associates with favorable disease outcome [65]. Our finding of its reduced expression in STCs *vs* LTCs is intriguing as it suggests that in glioblastoma too it may play a role similar to that performed in breast cancer, in the context of TGFβ-modulated pathways. Notably, also in the comparison of the ST peritumoral areas as opposed to the LT ones, we found some indications of a possible higher contribution of tumor “stromal” cells, as reactive astrocytes. IGFBP5 mRNA in fact, overexpressed in STPs vs LTPs, even if not previously described in the context of GBM, had been reported as expressed by reactive astrocytes supporting retinoblastoma growth [36]. By IHC, we were able to detect for the first time IGFBP5 in the cytoplasm and extracellular matrix of reactive astrocytes in C tissues from short term patients, and also in the cytoplasm of astrocytes present in STP samples, though with a lower intensity (Suppl. Fig. 4), while we did not find it in LT peritumoral regions (not shown). In addition, we found a sound overexpression of the extracellular protein lumican in LTPs as compared to ST ones, still corroborating the view of a great involvement of tumor microenvironment in the definition of long-term *vs* short-term survivors. This stromal protein has been very recently shown to positively correlate with prolonged survival after tumor resection in in pancreatic ductal adenocarcinomas, due to its limiting role in EGFR-expressing pancreatic cancer progression [66].

### A reduced A to G editing of miR-376c-3p distinguishes tumor centers from peritumor areas and is more evident in STCs compared to LTCs

When we considered the results of microRNA expression profiling, we were not able to find miRNAs clearly distinguishing STCs from LTCs in a statistically significant way (i.e. FDR < 0.05). However, we found a significant difference in the percentage of the A to G edited variants of miR-376c-3p: LTCs harbored, on average, more edited mir-376c-3p than STCs. In addition, we also found that this same miRNA is more edited in all Ps vs Cs. The A to G editing of miR-376c-3p had previously been described, specifically in brain, where adenosine deaminase acting on RNA-1, ADAR1, was shown to be the enzyme responsible of this specific editing [67]. Intriguingly, our SAGE data revealed a reduced expression of a brain-specific member of the adenosine deaminase gene family, ADARB2, in all Cs and Ps, of both ST and LT patients. Even if the editing activity of this enzyme has never been formally demonstrated, and an inhibitory role was rather proposed for ADARB2 [68], the concomitant reduction of miR-376c-3p editing and ADARB2 expression suggest a functional correlation between these two findings. A reduced editing of miR-376a, another member of the same miRNA cluster located on chromosome 14, and evolutionarily related to miR-376c, was previously reported in glioblastoma compared to healthy control tissues [69], and a functional consequence of this was identified in the differential targeting of specific mRNAs by the edited *vs* the non-edited miR-376a isoforms. In our case, we did not find any difference in the levels of miR-376a editing when comparing Cs vs Ps, or all samples vs healthy controls. However, the indication, obtained by target prediction, that some mRNAs highly relevant for tumor cell biology (e.g. CDKN1A and Drosha) could be differentially targeted by the non-edited, tumor enriched miR-376c isoform, is surely intriguing and deserves further investigation.

In conclusion, this comprehensive study of GBM tissues and peritumoral areas indicates some key molecules characterizing LT from ST tissues, and at the same time shows the commonality of some molecules and pathways (e.g. TGFβ) between tumor centers and peritumoral areas, strongly suggesting that such molecules/pathways should be targeted in both areas in order to fight, and defeat, glioblastoma.

## MATERIALS AND METHODS

### Tissue procurement and RNA extraction

In this study we used tissue samples coming from 13 GBM patients; 9 patients were classified as short-term survival and 4 cases as long-term survival (survival ≥ 36) (Table 1). All patients provided informed consent to use their tumor and peritumor material as well as clinical data for research purposes, none of them was identifiable. The study was approved by the local ethics committee (Catholic University Ethics Committee, Rome).

Tumor removal was achieved with resection margins that included the neighboring, apparently normal tissue (between 1 cm and 2 cm from the tumor border; larger resections were performed in tumors that grew far from eloquent areas) and the tumor, which were removed entirely *en bloc*.

Neuronavigation and intraoperative ultrasounds were used to maximize the extent of intracranial tumor resection. Thirty-five days after surgery (range, 30–40 days), patients received an external source limited-field radiotherapy (with an average dose of 60 grays administered in fractions). Simultaneously, chemotherapy with temozolomide (Temodal) was administered at a dose of 75 mg/m^2^ per day. After a 1-month break, patients received up to 6 cycles of temozolomide at a dose from 150 to 200 mg/m^2^ per day on the standard schedule of 5 days per week every 28 days. All patients underwent the same adjuvant therapy protocol.

All patients were followed as outpatients 1 month after surgery and every 3 months thereafter.

We obtained 13 pair samples from different areas: Tumor and Peritumor white matter tissues close to the tumor, between 1 cm and 2 cm from the tumor border. One series (tumor and peritumor tissues) was immediately fixed in 10% neutral buffered formalin for histological analysis and the second one was immediately frozen on dry ice for molecular analysis. All histological samples were reviewed by a board-certified neuropathologist and all tumors were classified as glioblastoma (WHO IV). Multiple levels of each paraffin block of samples used for research purposes (tumor and peritumor) were thoroughly examined. Specimens were histologically assessed using H&E sections. Neoplastic cells were identified by their nuclear atypia and heteropyknotic staining. Glial fibrillary acidic protein (GFAP) immunolabeling of peritumor area samples was used routinely to identify reactive astrocytes, which were identified by their clear, large eccentric nuclei; eosinophilic cytoplasm; and long, thick, stellate, GFAP-positive processes, according to Hoelzinger et al. [70].

Specimens from surrounding tumor area were assessed histologically by 2 independent pathologists who identified the presence of neoplastic cells in each sample. Two sections from each area were examined by 2 different observers independently, and at least 1000 to 2500 cells from 4 to 10 randomly selected fields in each section were counted. By using a light microscope (Axioskop 2 plus; Zeiss), the presence of tumor cells was evaluated at 3400X magnification. Samples with discrepant scoring were re-evaluated jointly on a second occasion, and agreement was reached. The results were categorized as ‘infiltrated’ if tumor cells were detected in at least 1 specimen. In the peritumoral areas classified as “non-infiltrated” in this study, no tumor cells were seen, and the expression of Ki-67, marker of proliferation detected by immunohistochemistry, was less than 1%. On the contrary, the peritumoral areas classified as “infiltrated”, showed a Ki-67 immunoreactivity of >> 1%.

CTRL specimens were derived from patients at the same ages operated for deep cavernomas with radiological signs of recent bleeding.

Total RNA was isolated from all tissue samples by RNA/DNA/Protein Purification Kit (Norgen, CA) as described by manufacturer's instructions followed by clean-up DNase digestion on column by RNase-Free DNase Kit (Norgen, CA).

### Western blot analysis

For Western blot analysis, total protein extract was isolated from tissue samples by RNA/DNA/Protein Purification Kit (Norgen, CA) as described by manufacturer's instructions. Equivalent amounts of protein extract were separated by electrophoresis on 12% SDS-PAGE gels and blotted onto nitrocellulose. The membranes were blocked with 5% nonfat dry milk and 0.1% Tween-20 in Phosphate-buffered saline and then incubated with antibodies followed by appropriate horseradish peroxidase-conjugated secondary antibody (Promega, USA). Rabbit polyclonal anti-LMTK3 (Abcam, UK) was used diluted 1:500, rabbit polyclonal anti-TGFBI (Thermo Scientific, USA) was diluted 1:1000, rabbit polyclonal anti-SOCS2 (Thermo Scientific, USA) was diluted 1:200. Rabbit polyclonal anti-β-actin (Sigma, USA) diluted 1:400 was used to reveal β-actin as a loading control.

### SAGE sequencing and analysis

SAGE barcoded libraries were prepared from 27 total RNA samples extracted independently from the cores and peritumoral regions of 13 glioblastoma tumors and 1 sample of healthy white matter. Each library was generated by using the SOLiD™ SAGE™ Kit with Barcoding Adaptor Module and a SOLiD™ RNA Barcoding Kit (Catalog nos. 4452811 and 4427046, Applied Biosystems, Foster City, CA), following the manufacturer's instructions. The barcoded libraries were combined and sequenced on 3 full slides on an Applied Biosystems SOLiD 4 System. Sequencing length was 35 bp. Library preparation, barcode addition, emulsion PCR, and SOLiD sequencing were performed at Genomnia Srl (Lainate, Milan, Italy).

Suppl. Table 3a summarizes all the samples and the target mapping percentages associated with this experiment. All the SAGE Color Space sequence files generated by the sequencing were corrected *de novo* for sequencing errors before mapping on the reference transcriptome, using the Life Technologies SAET (SOLiD Accuracy Enhancer Tool) version 2.2 program (https://www.biostars.org/static/downloads/solid/solid-denovo-assembly/saet.2.2/SAET.v2.2.pdf).

The error-corrected sequences were then trimmed to 27 nt from the original 35. These were mapped using the bowtie version 0.1.27 version 2 software against a reference 3′ UTR database derived from RefSeq and relative to the hg19 version of the human genome sequence. The alignments were parsed with ad-hoc created Perl scripts in order to extract and count the aligned SAGE tags beginning with the CATG sequence, with max 1 mismatch (globally) with the reference sequence and with a single mapping position on the reference sequence. The aligned reads counts have been then grouped by target RefSeq ID; analyzed for differential expression with the Bioconductor EdgeR library; annotated with a proprietary script.

The results consist in tables reporting the differentially expressed genes (by *P* value and False Discovery rate) for each of the comparisons which are summarized below.

STP vs STC; LTP vs LTC; LTP vs STP; LTC vs STC; STPs vs healthy control; LTPs vs healthy control; STCs vs healthy control; LTCs vs healthy control.

Single Sample Gene Set Enrichmemnt Analysis was performed using the SGEA module from the Gene Pattern Public Server (http://www.broadinstitute.org/cancer/software/genepattern) and the classified gene lists associated with the subtype calls from the expression values of the core TCGA samples (https://tcga-data.nci.nih.gov/docs/publications/gbm_exp/TCGA_unified_CORE_ClaNC840.txt).

### Feature selection with ReliefF

Weka 3.7 [71] was used to employ the the ReliefF algorithm [14]. ReliefF computes merit as the difference between the probability of observing a different value of a metric in a different class and the probability of observing a different value of a metric in the same class. Merit therefore simultaneously emphasized inter-class dishomogeneity and intra-class homogeneity (i.e. merit is high when an RNA is both different between different classes and equal between equal classes or in within the same class). The RNA molecules were then ranked according to merit, and the first 50 highest ranking molecules were selected for visual representation through a heatmap depicting the ratio of expression between classes for each RNA molecule. Mathematica 9 (R) was employed to generate the heatmaps.

### Microrna sequencing and analysis

A second aliquot of the same total RNA samples analyzed by SAGE sequencing was used to explore the small RNA fraction, after enrichment using the Invitrogen PureLink^®^miRNA Isolation Kit. The amount and quality of the enriched small RNA was examined using the Agilent Bioanalyzer. Barcoded sequencing libraries were generated from an average of 10 ng of purified small RNA using the Applied Biosystems SOLiD™ Small RNA library protocol (July 2011 Rev.B) with the Total RNA-Seq Kit (PN 4445374). The libraries were pooled and sequenced with the SOLiD 5500XL platform using 9 lanes. Sequence files in Colorspace format (.csfasta and .qual) included in the .xsq file were mapped and analysed using the ‘small RNA’ Lifetech Lifescope version 2.5.1 pipeline, using as targets the human genome GRCh37/hg19 and the dataset of mature sequences and precursors mirBase version 20 (June 2013). Matches with the genome repetitive elements (SINE, LINE..), snoRNAs, piRNAs, tRNAs and rRNAs were eliminated by the results through preliminary filtering. Sequence data are shown in Suppl. Table 3b. Filtered small RNA alignments in .bam format were converted in .fastq format and subjected to further analyses.

Read mapping and isomiRNA counts were made by using a stand-alone Perl program able to detect miRNA variants (isomiRNAs) in small RNA-Seq experiment [Grassi et. al. in preparation]. The program compares the sequenced reads with respect to canonical mature miRNA and relative premiRNA present in miRBase (version 20) [72]. For each expressed isomiRNA, the occurrences were counted and possibly grouped in order to define the corresponding miRNA counts. Subsequently, miRNA counts were normalized by using the TMM normalization procedure [73]. The samples were classified in two groups based on miRNA expression, according to the method described by Brunet et al. [74], by using the non-negative matrix factorization (NMF) R package [75]. In 10 patients, miRNA expression of C samples was compared with respect to that of the corresponding P samples. The differential expression analysis was made by using a generalized linear model (GLM) implemented in edgeR and able to handle the paired experimental design [76]. We considered for the analysis only the miRNAs with counts per million (cpm) > =10 in more than 4 (at least in one sample in the pair) of the 10 analyzed C-P pairs (469 in total). The derived *p*-values and the corresponding FDR values are reported in Suppl. Table 4. The comparison between the infiltrated P samples (7 samples) with non infiltrated P samples (3 samples) was made by using edgeR in the standard modality. Due to the reduced number of samples, we limited the analysis to the 464 miRNAs with at least 10 cpm in at least 3 samples.

Target genes of miR-376c-3p and its 6n AtoG edited variant were predicted by intersecting the results of the two programs miRanda [77] and PITA [78]; in both cases all the 3′UTRs of the Genecode genes derived by UCSC have been analyzed.

### Quantitative real-time-PCR

qRT-PCRs were performed to validate the expression of specific mature miRNAs, using pre-designed stem-loop primers (TaqMan MicroRNA Assay, Applied Biosystems-Life Technologies). 10 ng of total RNA isolated from tissue samples was used to generate cDNA by TaqMan MicroRNA Reverse Transcription Kit (Applied Biosystems-Life Technologies) according to the manufacturer's instructions.

For mRNA, 0.5 μg of total RNA isolated from tissue samples (pre-treated with DNase I) was used to generate cDNA by the SensiFAST cDNA Synthesis Kit (Bioline, London, UK) according to the manufacturer's instructions.

qRT-PCRs were conducted on an StepOnePlus Real-Time PCR System (Applied Biosystems-Life Technologies) using TaqMan Universal PCR Master Mix and the specific TaqMan^®^ Assays (probe and primer sets) (Applied Biosystems-Life Technologies). The small endogenous nuclear RNA U6 (RNU6B) and *TBP* were used as controls for normalization of mature miRNAs and mRNAs, respectively. The relative amount of each substrate was calculated by the 2^−ΔΔCT^ method [79]. All the primers were supplied by Applied Biosystems: CA3, ID Hs00193123_m1; BCAR3, ID Hs00981962_m1; ENPP2, ID Hs00905117_m1; CXCL14, ID Hs01557413_m1; TOP2, ID Hs01032137_m1; COL1A2, ID Hs00164099_m1; MSR1, ID Hs00234007_m1; ADARB2, ID Hs00218878_m1; LPHN2, ID Hs00202347_m1; NES, ID Hs04187831_g1; H19, ID Hs00262142_g1; LINC00320, ID Hs01373561_m1; TBP, ID Hs99999910_m1; miR-338-3p, ID 002252; miR-584-5p, ID 001624; miR-1290, ID 002863; RNU6B, ID 001093; miR-34c-5p, ID 000428; miR-23b, ID 000400; miR-590-5p, ID 001984; miR-219a-5p, ID 00522; miR-340-5p, ID 002258.

### Immunohistochemistry

Tissue samples were fixed in 4% formalin and embedded in paraffin. Two-μm thick serial sections were cut for each case; sections were mounted on poly-L-lysine coated glass slides (X-tra^®^ Adhesive slides, Leica Biosystems, Wetzlar, Germany) and dried overnight at 37°C followed by 1 h at 60°C.

Briefly, sections were deparaffinized, hydrated and antigen was retrieved with Heat-Induced Epitope Retrieval (HIER) and pH 6 within the Dako PT Link instrument with a highly stabilized retrieval system (PT-link, Dako, Glostrup, Denmark) except for GFAP that does not require retrieval. All immunohistochemical stainings were performed using UltraTek HRP Anti-Polyvalent, developed with 3-3′diaminobenzidine, DAB Chromogen/Substrate Kit (ScyTek Laboratories, South 600 West, Logan, USA) and counterstained with Harris' Haematoxylin. In particular, endogenous peroxidase was blocked with Peroxide Block for 8 min, sections were incubated 20 min with Avidin/Biotin Blocking System (Spring Bioscience, Pleasanton, CA) and then with primary antibodies for 30 min at room temperature. In detail, CA3 [4A12-1A3], 1:200 dilution, and IGFBP5 [MM0344-8D21] 1:50 dilution, (Novus Biologicals, Southpark Way, Littleton, CO, USA); CXCL14, 1:50 dilution and TGFBI, 1:100 dilution, (Thermo Fisher Waltham, MA, USA); CD44, 1:100 dilution and GFAP, 1:200 dilution, (Spring Bioscience, Pleasanton, CA); CD68 Monoclonal Mouse Anti-Human CD68, Clone PG-M1, 1:200 dilution, (Dako, Glostrup, Denmark), were used. Antibodies specific staining patterns were observed qualitatively. IHC evaluation was performed by a Leica BM5000 optical microscope, and Leica DMD108 digital microimaging sytem (Leica Microsystems; Wetzlar, Germany) was used to acquire images.

## SUPPLEMENTARY FIGURES AND TABLES




