# Supplementary material for: The transcriptome and miRNome profiling of glioblastoma tissues and peritumoral regions highlights molecular pathways shared by tumors and surrounding areas and reveals differences between short-term and long-term survivors
Source: Oncotarget. 2015 Jun 1;6(26):22526–52. doi: 10.18632/oncotarget.4151 (PMC4673180; doi:10.18632/oncotarget.4151)
Supplement: Supplementary file 1 [file oncotarget-06-22526-s001.pdf]

SUPPLEMENTARY FIGURES AND TABLES

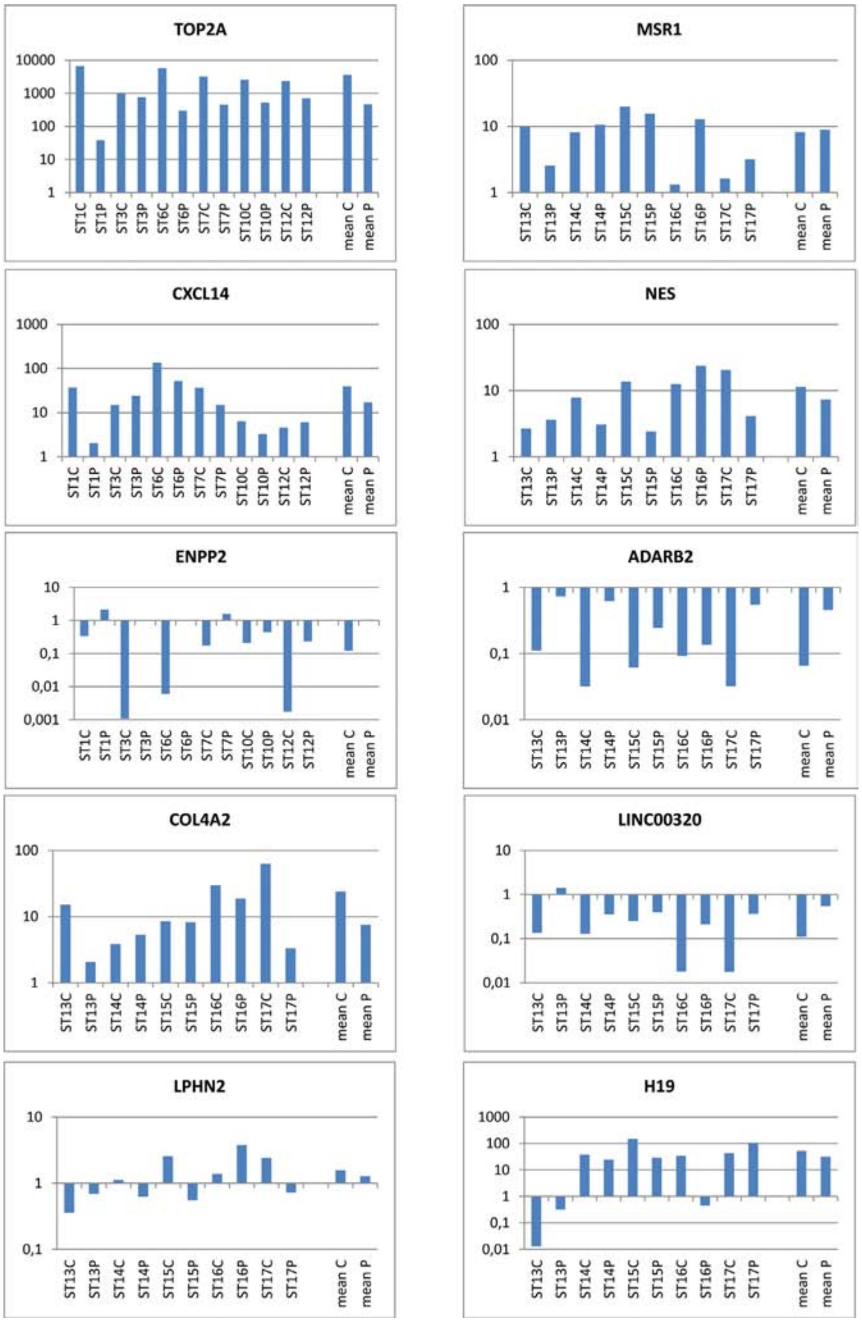

Supplementary Figure S1: RT-qPCR validation of mRNA expression in STCs and STPs vs heathy white matter. Values are all presented in a Log_{10}-based scale with reference to healthy white matter expression, posed = 1, and were calculated using the expression of TBP as the housekeeping normalizer. For each mRNA, expression values of both single samples and average values for STCs and STPs are displayed. ST7, ST10 and ST12 are samples previously analyzed by SAGE, while ST1, ST3, ST6, ST13, ST14, ST15, ST16, and ST17 are new samples.

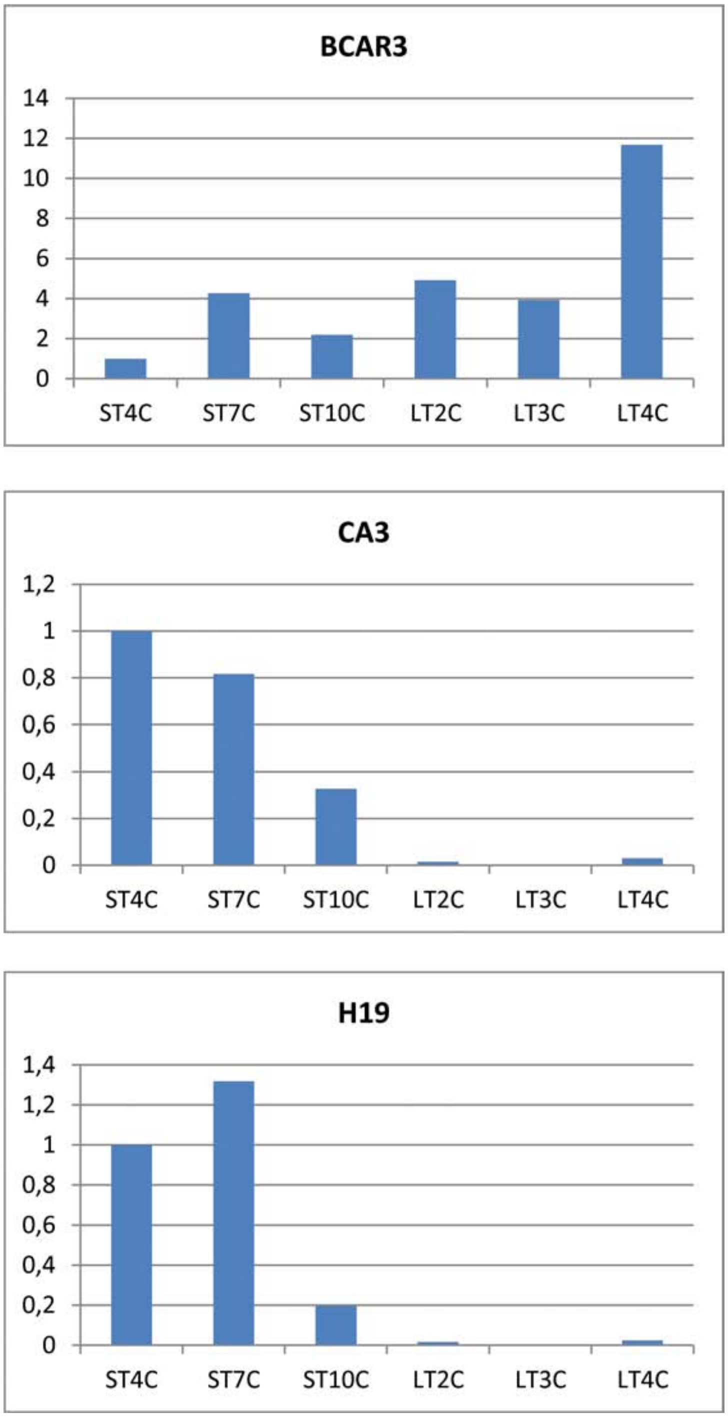

Supplementary Figure S2: RT-qPCR validation of mRNA expression in STC and LTC samples. For all three mRNAs, the expression measured in sample ST4C is arbitrarily posed = 1 and the values in the other samples are expressed with reference to ST4C. Values were calculated using the expression of TBP as the housekeeping normalizer.

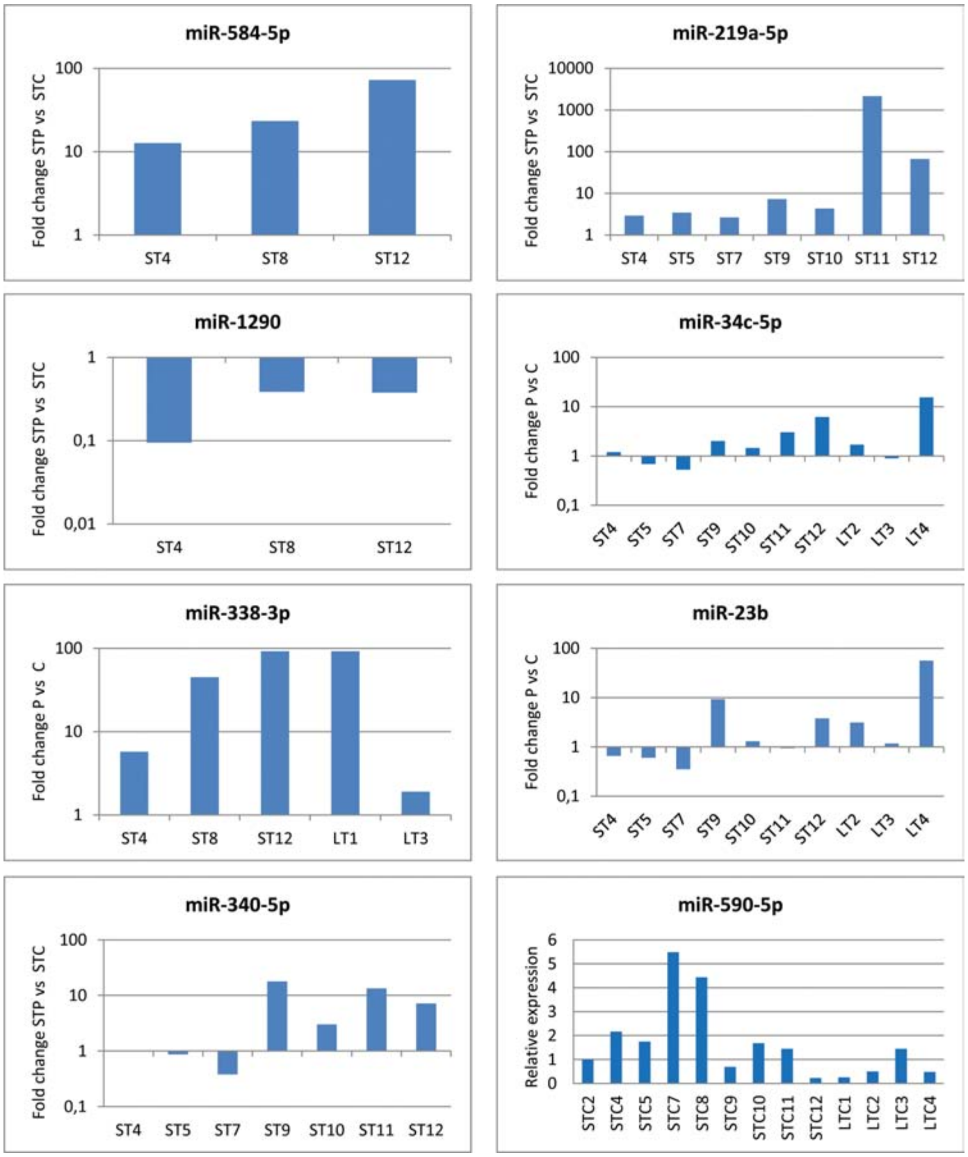

Supplementary Figure S3: RT-qPCR validation of miRNA differential expression. In all panels except that of miR-590–5p, values are presented in a Log_{10}-based scale and represent the relative expression of each P sample compared to its own C sample. For miR-590–5p, only values for C samples are shown, and the expression of all samples is referred to that of ST2C, arbitrarily posed = 1, in a linear scale. Transcript levels were normalized by U6 expression. ST4, ST7, ST8, ST10, ST11, ST12, LT1, LT2, and LT3 are samples previously analyzed by deep-sequencing, while ST5, ST9 and LT4 did not undergo previous miRNA sequencing.

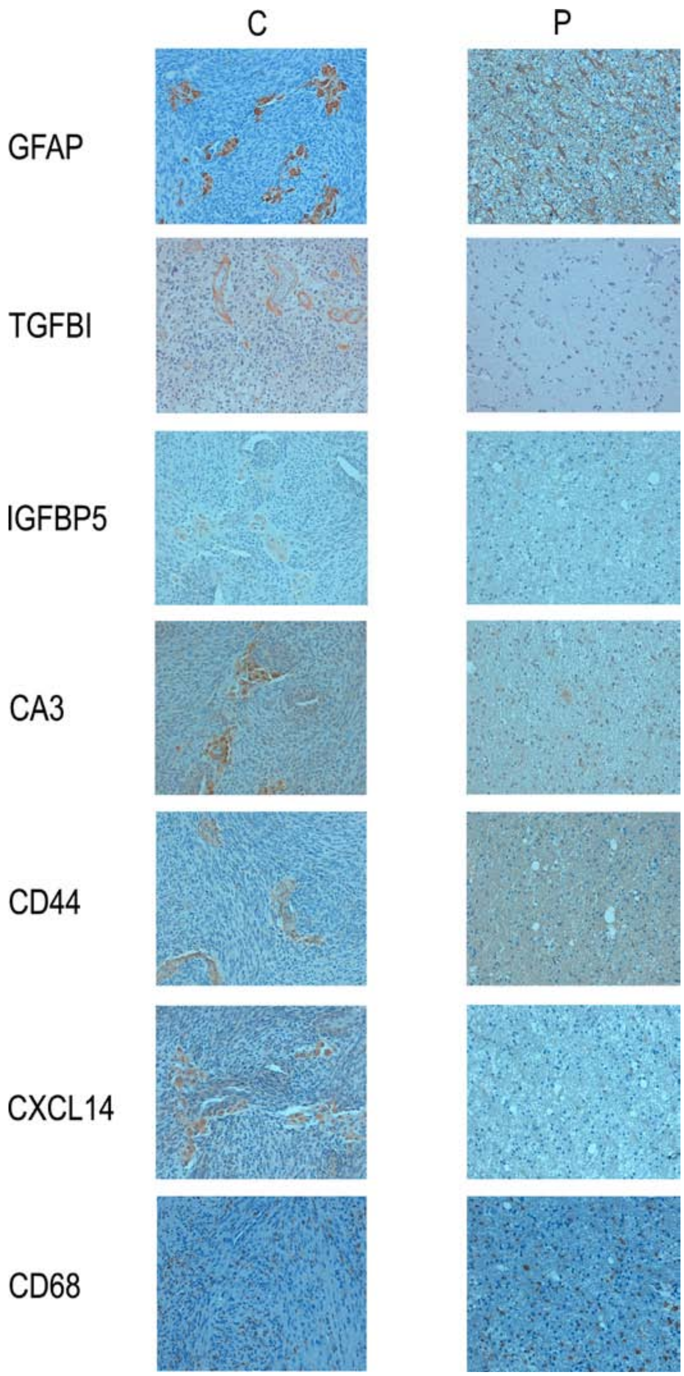

Supplementary Figure S4: Representative images of IHC staining of C and P samples from ST patients. Magnification = 20X. Beside specific markers discussed in this work, the staining for GFAP and CD68 is shown, as a measure of the presence of reactive astrocytes and microglia/macrophages, respectively.

Supplementary Table S1a. The highlighted genes are those in common between ST and LT P vs C comparisons

STP vs STC

NCBI RefSeq	logFC	*P* Value	FDR	gene name
NM_002147	−5.569394	0.000055	0.012877	HOXB5
NM_001136030	−5.264828	0.000000	0.000435	KIAA0748
NR_002196	−5.058820	0.000000	0.000076	H19
NM_020346	−4.741178	0.000000	0.000261	SLC17A6
NM_207366	−4.575584	0.000002	0.002817	SEPT14
NM_052947	−4.344015	0.000023	0.008316	ALPK2
NM_015419	−4.155425	0.000005	0.003898	MXRA5
NM_004209	−4.124442	0.000025	0.008471	SYNGR3
NM_152701	−4.057768	0.000091	0.016850	ABCA13
NM_001958	−3.946988	0.000395	0.038386	EEF1A2
NM_002345	−3.779973	0.000005	0.003898	LUM
NM_080617	−3.771591	0.000000	0.001221	CBLN4
NM_003063	−3.769110	0.000156	0.024136	SLN
NM_206963	−3.698713	0.000009	0.004814	RARRES1
NM_003385	−3.547955	0.000356	0.036845	VSNL1
NM_000129	−3.523695	0.000090	0.016850	F13A1
NM_001112741	−3.503232	0.000245	0.030715	KCNC1
NM_000088	−3.258647	0.000087	0.016711	COL1A1
NM_030885	−3.222203	0.000302	0.034155	MAP4
NM_015464	−3.125329	0.000511	0.045544	SOSTDC1
NM_002010	−3.124320	0.000388	0.038317	FGF9
NM_021076	−3.116797	0.000323	0.035601	NEFH
NM_001849	−3.054699	0.000045	0.011257	COL6A2
NM_005328	−3.032581	0.000024	0.008316	HAS2
NM_001195259	−3.024605	0.000424	0.040287	LOC100507588
NM_006308	−3.003269	0.000110	0.019241	HSPB3
NM_000089	−2.963589	0.000065	0.014417	COL1A2
NM_006528	−2.941053	0.000043	0.011213	TFPI2
NM_000093	−2.861029	0.000002	0.002817	COL5A1
NM_015052	−2.799206	0.000196	0.026836	HECW1
NM_002178	−2.737723	0.000071	0.014983	IGFBP6
NM_007224	−2.672742	0.000050	0.012037	NXPH4
NM_198993	−2.662271	0.000015	0.006717	STAC2
NM_014279	−2.607944	0.000119	0.020211	OLFM1
NM_006855	−2.604514	0.000321	0.035601	KDELR3

(*Continued*)

STP vs STC				
**NCBI RefSeq**	**logFC**	***P* Value**	**FDR**	**gene name**
NM_002523	−2.571896	0.000226	0.028610	NPTX2
NM_001099334	−2.474862	0.000301	0.034155	C2orf80
NM_005297	−2.470275	0.000227	0.028639	MCHR1
NM_015187	−2.431113	0.000005	0.003832	SEL1L3
NM_001010927	−2.422709	0.000096	0.017339	TIAM2
NM_002023	−2.421199	0.000545	0.046514	FMOD
NM_032785	−2.349596	0.000566	0.047421	AGBL4
NM_001962	−2.337194	0.000032	0.009627	EFNA5
NM_133468	−2.328038	0.000032	0.009627	BMPER
NM_001080467	−2.318882	0.000031	0.009627	MYO5B
NM_004976	−2.207445	0.000501	0.045267	KCNC1
NM_052899	−2.196373	0.000293	0.034078	GPRIN1
NM_020939	−2.161106	0.000164	0.024483	CPNE5
NM_152774	−2.121101	0.000478	0.043523	TMEM196
NM_173567	−2.115181	0.000264	0.032688	EPHX4
NM_023940	−2.007290	0.000264	0.032688	RASL11B
NM_006818	−1.995112	0.000183	0.025647	MLLT11
NM_021020	−1.969728	0.000518	0.045544	LZTS1
NM_000474	−1.961860	0.000337	0.035986	TWIST1
NM_012419	−1.958399	0.000039	0.010711	RGS17
NM_000313	−1.923526	0.000348	0.036561	PROS1
NM_001661	−1.890931	0.000086	0.016711	ARL4D
NM_152308	−1.871535	0.000328	0.035775	RMI2
NM_173582	−1.842811	0.000316	0.035233	PGM2L1
NM_080927	−1.821366	0.000294	0.034078	DCBLD2
NM_001848	−1.816115	0.000550	0.046545	COL6A1
NM_031455	−1.794091	0.000426	0.040287	CCDC3
NM_002081	−1.787275	0.000022	0.008239	GPC1
NM_139055	−1.749625	0.000408	0.039279	ADAMTS15
NM_018110	−1.722781	0.000446	0.041259	DOK4
NM_152745	−1.716040	0.000394	0.038386	NXPH1
NM_002254	−1.688398	0.000211	0.027793	KIF3C
NM_018159	−1.571331	0.000201	0.027359	NUDT11
NM_001029858	−1.568188	0.000443	0.041259	SLC35F1
NM_153367	1.423345	0.000071	0.014983	ZCCHC24

(*Continued*)

STP vs STC				
NCBI RefSeq	logFC	*P* Value	FDR	gene name
NM_001128228	1.468162	0.000518	0.045544	TPRN
NM_013995	1.522396	0.000128	0.021051	LAMP2
NM_000097	1.527156	0.000175	0.025115	CPOX
NR_033258	1.531945	0.000604	0.049716	ZEB2
NM_207342	1.539587	0.000534	0.046379	STEAP1B
NM_004232	1.553772	0.000330	0.035790	SOCS6
NM_002163	1.621962	0.000389	0.038317	IRF8
NM_007170	1.630647	0.000068	0.014755	TESK2
NM_017831	1.640681	0.000404	0.039110	RNF125
NM_181809	1.646801	0.000280	0.033486	BMP8A
NM_006820	1.651685	0.000569	0.047565	IFI44L
NM_015254	1.676724	0.000347	0.036561	KIF13B
NM_021205	1.677523	0.000160	0.024402	RHOU
NM_052905	1.681206	0.000175	0.025115	FMNL2
NM_148893	1.694634	0.000097	0.017362	SVIP
NM_024761	1.703754	0.000116	0.019898	MOB3B
NM_000891	1.737725	0.000166	0.024576	KCNJ2
NM_007168	1.739964	0.000145	0.023071	ABCA8
NR_028038	1.770627	0.000280	0.033486	GATS
NM_005028	1.772968	0.000265	0.032688	PIP4K2A
NM_021202	1.791188	0.000095	0.017289	TP53INP2
NM_207578	1.823209	0.000586	0.048767	PRKACB
NM_024871	1.834248	0.000010	0.005100	MAP6D1
NM_017762	1.840787	0.000388	0.038317	MTMR10
NM_000271	1.842677	0.000075	0.015356	NPC1
NM_005099	1.872770	0.000044	0.011213	ADAMTS4
NM_001085486	1.889470	0.000212	0.027793	SEPP1
NM_001093726	1.889786	0.000195	0.026836	SEPP1
NM_004140	1.895692	0.000033	0.009627	LLGL1
NM_005410	1.903017	0.000163	0.024418	SEPP1
NR_037595	1.917627	0.000371	0.037586	LOC646576
NM_020698	1.924270	0.000039	0.010711	TMCC3
NM_012113	1.932122	0.000020	0.008002	CA14
NM_152588	1.933588	0.000101	0.017868	TMTC2
NM_133477	1.952816	0.000019	0.007861	SYNPO2

(*Continued*)

STP vs STC				
NCBI RefSeq	logFC	*P* Value	FDR	gene name
NM_001101387	1.984942	0.000003	0.002818	PIRT
NM_018342	2.045989	0.000045	0.011213	TMEM144
NM_014442	2.066763	0.000086	0.016711	SIGLEC8
NM_001001669	2.080482	0.000091	0.016850	ARHGEF37
NR_045555	2.091795	0.000338	0.035986	ENPP2
NM_181789	2.121969	0.000377	0.037834	GLDN
NR_024100	2.132853	0.000113	0.019672	LINC00323
NM_031453	2.143295	0.000039	0.010711	FAM107B
NM_175922	2.149763	0.000013	0.006267	PRR18
NM_006272	2.162147	0.000005	0.003898	S100B
NM_001004298	2.197380	0.000216	0.027992	C10orf90
NM_032515	2.207265	0.000011	0.005352	BOK
NM_002612	2.219324	0.000180	0.025569	PDK4
NM_001162995	2.232474	0.000030	0.009627	C17orf109
NM_002984	2.246163	0.000447	0.041259	CCL4
NM_015549	2.247016	0.000513	0.045544	PLEKHG3
NM_024577	2.251457	0.000022	0.008239	SH3TC2
NR_046117	2.269716	0.000004	0.003147	ELOVL1
NM_199355	2.290626	0.000491	0.044541	ADAMTS18
NM_006557	2.320626	0.000153	0.024020	DMRT2
NM_016235	2.323020	0.000025	0.008471	GPRC5B
NM_033133	2.332784	0.000032	0.009627	CNP
NR_033862	2.362308	0.000014	0.006457	DKFZp451B082
NM_181712	2.363229	0.000001	0.002474	KANK4
NM_021979	2.411617	0.000000	0.000696	HSPA2
NM_001063	2.450865	0.000003	0.003074	TF
NM_015194	2.524840	0.000010	0.005100	MYO1D
NM_020459	2.545756	0.000120	0.020262	PAIP2B
NM_015993	2.579223	0.000041	0.010854	PLLP
NM_182623	2.584754	0.000054	0.012780	FAM131C
NM_018685	2.618145	0.000002	0.002600	ANLN
NM_182935	2.636373	0.000376	0.037834	MOBP
NM_153034	2.682167	0.000310	0.034710	ZNF488
NM_173084	2.704189	0.000001	0.001408	TRIM59

(*Continued*)

STP vs STC				
NCBI RefSeq	logFC	*P* Value	FDR	gene name
NM_020435	2.706738	0.000007	0.004299	GJC2
NM_198406	2.767860	0.000051	0.012128	PAQR6
NM_005076	2.783902	0.000005	0.003832	CNTN2
NM_001142287	2.834277	0.000002	0.002817	SEMA4D
NM_016533	2.874302	0.000004	0.003147	NINJ2
NM_020715	2.912580	0.000001	0.001576	PLEKHH1
NM_012128	2.923552	0.000293	0.034078	CLCA4
NM_144626	2.948181	0.000087	0.016711	TMEM125
NM_003657	2.987106	0.000007	0.004351	BCAS1
NM_001252511	3.077118	0.000551	0.046545	SOAT1
NM_014717	3.121805	0.000021	0.008185	ZNF536
NM_024306	3.133304	0.000022	0.008239	FA2H
NR_024090	3.134724	0.000007	0.004272	LINC00320
NM_001252512	3.187203	0.000278	0.033486	SOAT1
NM_014698	3.187544	0.000047	0.011594	TMEM63A
NM_004525	3.267668	0.000003	0.003074	LRP2
NR_037155	3.320605	0.000014	0.006513	SEPT4
NM_003101	3.438315	0.000071	0.014983	SOAT1
NM_012276	3.668811	0.000001	0.001576	LILRA4
NM_001757	4.067284	0.000007	0.004272	CBR1
NM_012125	4.082389	0.000008	0.004691	CHRM5
NM_178172	4.423640	0.000000	0.000115	GPIHBP1
NM_001015049	4.759540	0.000173	0.025102	BAG5
NM_004873	4.988192	0.000068	0.014755	BAG5
NM_000371	5.045914	0.000044	0.011213	TTR
NM_001015048	5.494646	0.000007	0.004224	BAG5

Supplementary Table S1b. The highlighted genes are those in common between ST and LT P vs C comparisons

LTP vs LTC				
NCBI Ref seq	gene name	logFC	*P* Value	FDR
NM_003385	VSNL1	−5.77575	0.00000	0.00237
NM_144967	ARHGAP36	−5.33704	0.00000	0.00044
NM_005458	GABBR2	−4.98110	0.00000	0.00003
NM_153836	CREG2	−4.39906	0.00004	0.00727
NM_178539	FAM19A2	−4.21712	0.00002	0.00619
NR_024281	LOC157627	−4.00905	0.00029	0.02957
NM_152447	LRFN5	−3.71344	0.00035	0.03237
NM_001137560	TMEM151B	−3.68679	0.00040	0.03630
NM_015576	ERC2	−3.57882	0.00003	0.00727
NM_006317	BASP1	−3.53838	0.00005	0.00929
NM_044472	CDC42	−3.53243	0.00010	0.01414
NM_001040272	ADAMTSL1	−3.52433	0.00013	0.01747
NM_003026	SH3GL2	−3.50989	0.00034	0.03237
NM_006931	SLC2A3	−3.43415	0.00001	0.00316
NM_023004	RTN4R	−3.35136	0.00007	0.01037
NM_030965	ST6GALNAC5	−3.18779	0.00048	0.04062
NM_014632	MICAL2	−3.15741	0.00056	0.04495
NM_173582	PGM2L1	−3.09246	0.00003	0.00727
NM_198993	STAC2	−3.07060	0.00057	0.04495
NM_001031701	NT5DC3	−3.02988	0.00032	0.03184
NM_016588	NRN1	−2.98659	0.00004	0.00820
NM_001554	CYR61	−2.96192	0.00006	0.00936
NM_005809	PRDX2	−2.94262	0.00035	0.03237
NM_001110199	SRRM3	−2.77553	0.00065	0.04992
NM_006818	MLLT11	−2.58536	0.00059	0.04659
NM_016533	NINJ2	2.51378	0.00056	0.04495
NM_007168	ABCA8	2.55726	0.00045	0.03917
NM_005028	PIP4K2A	2.67217	0.00063	0.04904
NM_181789	GLDN	2.69397	0.00046	0.03917
NM_001162995	C17orf109	2.80857	0.00019	0.02276
NM_020717	SHROOM4	2.90007	0.00014	0.01874
NM_001982	ERBB3	2.90248	0.00027	0.02793
NM_015993	PLLP	2.90935	0.00021	0.02400

(*Continued*)

LTP vs LTC				
NCBI Ref seq	gene name	logFC	*P* Value	FDR
NM_032515	BOK	2.92561	0.00015	0.01917
NM_024871	MAP6D1	2.93555	0.00030	0.02957
NM_024577	SH3TC2	2.93835	0.00035	0.03237
NM_006557	DMRT2	2.99564	0.00023	0.02492
NM_033133	CNP	3.00237	0.00041	0.03638
NM_020715	PLEKHH1	3.23699	0.00006	0.00936
NM_001142287	SEMA4D	3.29139	0.00027	0.02793
NM_001063	TF	3.32732	0.00008	0.01108
NM_006941	SOX10	3.34967	0.00049	0.04080
NM_005099	ADAMTS4	3.35816	0.00003	0.00727
NM_001853	COL9A3	3.36572	0.00001	0.00269
NM_021202	TP53INP2	3.39829	0.00005	0.00927
NR_002798	NAPSB	3.43986	0.00001	0.00237
NM_016229	CYB5R2	3.45520	0.00003	0.00727
NM_175922	PRR18	3.51599	0.00004	0.00820
NM_018342	TMEM144	3.59207	0.00016	0.02010
NM_020435	GJC2	3.61790	0.00001	0.00237
NM_021979	HSPA2	3.64306	0.00003	0.00727
NM_012113	CA14	3.65241	0.00000	0.00237
NM_005076	CNTN2	3.66118	0.00005	0.00908
NR_045555	ENPP2	3.67276	0.00006	0.00936
NM_080865	GPR62	3.70338	0.00022	0.02446
NM_024306	FA2H	3.75565	0.00006	0.00936
NM_018685	ANLN	3.83841	0.00000	0.00237
NM_178172	GPIHBP1	3.85776	0.00000	0.00237
NM_144626	TMEM125	3.88994	0.00019	0.02276
NM_003657	BCAS1	3.90917	0.00008	0.01161
NM_198406	PAQR6	4.07819	0.00000	0.00237
NM_012128	CLCA4	4.10237	0.00004	0.00727
NM_014698	TMEM63A	4.11341	0.00000	0.00129
NM_080658	ACY3	4.30452	0.00004	0.00727
NM_005276	GPD1	4.53908	0.00001	0.00390
NM_004525	LRP2	4.60256	0.00001	0.00269
NM_001757	CBR1	4.87779	0.00000	0.00191
NM_012276	LILRA4	4.98385	0.00000	0.00044
NM_182935	MOBP	5.20110	0.00000	0.00211

Supplementary Table S2a. Functional Annotation Clustering of predicted targets of miR-376c edited only. Listed are the four top-ranking annotation clusters, characterized by Terms with a p (modified Fisher Exact *P*-Value) < 0.05. The "Count" column indicates the number of genes involved in the relative enriched term.

Annotation Cluster 1	Enrichment Score: 1.8275		
**Category**	**Term**	**Count**	***P* Value**
GOTERM_CC_FAT	GO:0030135~coated vesicle	14	0.00014
GOTERM_CC_FAT	GO:0016023~cytoplasmic membrane-bounded vesicle	27	0.001
GOTERM_CC_FAT	GO:0030136~clathrin-coated vesicle	11	0.0015
GOTERM_CC_FAT	GO:0031988~membrane-bounded vesicle	27	0.0016
GOTERM_CC_FAT	GO:0030659~cytoplasmic vesicle membrane	10	0.0070
GOTERM_CC_FAT	GO:0031410~cytoplasmic vesicle	27	0.0079
GOTERM_CC_FAT	GO:0012506~vesicle membrane	10	0.0118
GOTERM_CC_FAT	GO:0031982~vesicle	27	0.0131
GOTERM_CC_FAT	GO:0044433~cytoplasmic vesicle part	11	0.0166
GOTERM_CC_FAT	GO:0008021~synaptic vesicle	5	0.1148
**Annotation Cluster 2**	**Enrichment Score: 1.9777**		
**Category**	**Term**	**Count**	***P* Value**
KEGG_PATHWAY	hsa04340:Hedgehog signaling pathway	7	0.0025
KEGG_PATHWAY	hsa04310:Wnt signaling pathway	11	0.0042
GOTERM_BP_FAT	GO:0016055~Wnt receptor signaling pathway	10	0.0092
KEGG_PATHWAY	hsa04916:Melanogenesis	8	0.0114
KEGG_PATHWAY	hsa05217:Basal cell carcinoma	6	0.0115
GOTERM_BP_FAT	GO:0007223~Wnt receptor signaling pathway, calcium modulating pathway	3	0.1065
**Annotation Cluster 3**	**Enrichment Score: 1.9749**		
**Category**	**Term**	**Count**	***P* Value**
GOTERM_MF_FAT	GO:0008270~zinc ion binding	85	0.0014
GOTERM_MF_FAT	GO:0046914~transition metal ion binding	93	0.0133
GOTERM_MF_FAT	GO:0046872~metal ion binding	131	0.0147
GOTERM_MF_FAT	GO:0043169~cation binding	131	0.0199
GOTERM_MF_FAT	GO:0043167~ion binding	132	0.0243

Supplementary Table S2b. Functional Annotation Clustering of predicted targets of miR-376c non-edited only. Listed are the four top-ranking annotation clusters, characterized by Terms with a p (modified Fisher Exact *P*-Value) < 0.05. The "Count" column indicates the number of genes involved in the relative enriched term.

**Annotation Cluster 1**	**Enrichment Score: 1.8275**		
**Category**	**Term**	**Count**	***P* Value**
GOTERM_MF_FAT	GO:0051119~sugar transmembrane transporter activity	4	0.0015
GOTERM_MF_FAT	GO:0005355~glucose transmembrane transporter activity	3	0.0074
GOTERM_MF_FAT	GO:0015149~hexose transmembrane transporter activity	3	0.0107
GOTERM_MF_FAT	GO:0015145~monosaccharide transmembrane transporter activity	3	0.0119
GOTERM_BP_FAT	GO:0008643~carbohydrate transport	4	0.0184
GOTERM_BP_FAT	GO:0055085~transmembrane transport	7	0.4227
**Annotation Cluster 2**	**Enrichment Score: 1.8031**		
**Category**	**Term**	**Count**	***P* Value**
GOTERM_MF_FAT	GO:0051119~sugar transmembrane transporter activity	4	0.0015
GOTERM_MF_FAT	GO:0005402~cation:sugar symporter activity	3	0.0085
GOTERM_MF_FAT	GO:0005351~sugar:hydrogen symporter activity	3	0.0085
GOTERM_BP_FAT	GO:0008643~carbohydrate transport	4	0.01839
GOTERM_MF_FAT	GO:0015295~solute:hydrogen symporter activity	3	0.0201
GOTERM_MF_FAT	GO:0015294~solute:cation symporter activity	4	0.0510
GOTERM_MF_FAT	GO:0015293~symporter activity	4	0.1195
**Annotation Cluster 3**	**Enrichment Score: 1.6909**		
**Category**	**Term**	**Count**	***P* Value**
GOTERM_BP_FAT	GO:0009952~anterior/posterior pattern formation	7	0.0018
GOTERM_BP_FAT	GO:0001822~kidney development	4	0.0581
GOTERM_BP_FAT	GO:0001655~urogenital system development	4	0.0802

(*Continued*)

Annotation Cluster 4	Enrichment Score: 1.6062		
**Category**	**Term**	**Count**	***P* Value**
GOTERM_BP_FAT	GO:0043066~negative regulation of apoptosis	11	0.0015
GOTERM_BP_FAT	GO:0043069~negative regulation of programmed cell death	11	0.0017
GOTERM_BP_FAT	GO:0060548~negative regulation of cell death	11	0.0017
GOTERM_BP_FAT	GO:0042981~regulation of apoptosis	16	0.0067
GOTERM_BP_FAT	GO:0043067~regulation of programmed cell death	16	0.0073
GOTERM_BP_FAT	GO:0010941~regulation of cell death	16	0.0076
GOTERM_BP_FAT	GO:0006916~anti-apoptosis	5	0.1210
GOTERM_BP_FAT	GO:0006917~induction of apoptosis	6	0.1707
GOTERM_BP_FAT	GO:0012502~induction of programmed cell death	6	0.1722
GOTERM_BP_FAT	GO:0043065~positive regulation of apoptosis	7	0.2023
GOTERM_BP_FAT	GO:0043068~positive regulation of programmed cell death	7	0.2066
GOTERM_BP_FAT	GO:0010942~positive regulation of cell death	7	0.2095

Supplementary Table S3a. SAGE mapping data

Sample		Total Reads	Mapped Reads	Pct
LT1	Core	49,880,749	9,892,252	19.83
LT1	Periphery	39,457,660	6,995,892	17.73
LT2	Core	48,964,296	9,608,297	19.62
LT2	Periphery	41,719,818	10,178,915	24.40
LT3	Core	52,433,392	10,727,457	20.46
LT3	Periphery	39,458,154	8,488,864	21.51
LT4	Core	45,988,520	9,276,126	20.17
LT4	Periphery	42,564,349	7,761,274	18.23
ST2	Core	46,246,926	7,259,364	15.70
ST2	Periphery	34,777,045	6,507,229	18.71
ST4	Core	40,054,931	9,030,532	22.55
ST4	Periphery	39,798,188	5,494,460	13.81
ST5	Core	48,285,130	15,849,600	32.83
ST5	Periphery	48,007,450	10,476,478	21.82
ST7	Core	41,336,824	9,385,032	22.70
ST7	Periphery	35,508,090	5,907,134	16.64
ST8	Core	50,496,900	10,874,303	21.53
ST8	Periphery	40,965,072	8,436,507	20.59
ST9	Core	45,707,533	9,205,826	20.14
ST9	Periphery	38,299,947	8,189,830	21.38
ST10	Core	49,345,681	10,884,536	22.06
ST10	Periphery	43,852,579	8,950,219	20.41
ST11	Core	55,762,945	19,243,136	34.51
ST11	Periphery	259,944,467	67,896,275	26.12
ST12	Core	50,982,086	17,454,114	34.24
ST12	Periphery	54,881,420	17,709,336	32.27
White matter		35,305,044	6,830,039	19.35

Supplementary Table S3b. microRNA mapping data

Sample		Sequenced Reads	ReadsMapped onto genome	ReadsMapped onto miRBase 20
LT1	Core	44,151,333	29,433,911	20,275,943
LT1	Periphery	44,703,697	18,328,838	11,546,461
LT2	Core	37,720,303	19,065,877	8,713,172
LT2	Periphery	44,306,549	20,245,773	8,055,664
LT3	Core	63,464,393	34,661,519	12,730,728
LT3	Periphery	51,482,767	28,682,529	15, 769, 018
ST2	Core	43,075,651	21,041,870	9,803,832
ST2	Periphery	39,880,317	26,718,015	9,012,741
ST4	Core	53,652,314	28,309,480	18,945,949
ST4	Periphery	51,185,318	21,628,643	10, 369, 993
ST7	Core	39,112,199	15,142,288	10,109,371
ST7	Periphery	31,181,903	18,579,603	11,877,785
ST8	Core	33,542,938	18,709,721	10,198,371
ST8	Periphery	30,924,127	15,395,905	10,154,977
ST10	Core	32,555,595	18,316,048	9,958,965
ST10	Periphery	51,023,372	25,279,892	9,884,276
ST11	Core	36,085,311	21,198,037	12,357,742
ST11	Periphery	35,142,083	18,703,494	11,913,730
ST12	Core	40,449,643	10,054,210	2,747,734
ST12	Periphery	48,362,074	17,394,093	8,288,585
White matter		38,848,422	22,195,255	11,640,032

Supplementary Table S4. MiRNA differential expression analysis. We tested differential expressed miRNAs in 10 patients by using a generalized linear model able to handle the paired experimental design. For each miRNA, the table reports the fold change (in log scale), counts per million (in log scale), likelihood ratio (LR), ***p***-value, false discovery rate (FDR) and normalized counts in each sample and in the healthy white matter sample (SB).

Supplementary Table S5. Characteristics of patients analyzed for RT-qPCR validation of deep sequencing data as shown in Suppl Figure 1. ST15 represents an unclear result as for the presence of infiltrating tumor cells in the peritumoral area.

Patient	Sex	Age	Survival (months)	Peritumoral infiltration
**ST1**	M	60	12	no
**ST3**	M	65	11	yes
**ST6**	M	76	15	no
**ST13**	F	71	13	no
**ST14**	M	71	33	yes
**ST15**	F	59	9	unclear
**ST16**	M	67	12	no
**ST17**	M	37	4	yes
